# Strengthening referral systems in community health programs: a qualitative study in two rural districts of Maputo Province, Mozambique

**DOI:** 10.1186/s12913-019-4076-3

**Published:** 2019-04-29

**Authors:** Celso Give, Sozinho Ndima, Rosalind Steege, Hermen Ormel, Rosalind McCollum, Sally Theobald, Miriam Taegtmeyer, Maryse Kok, Mohsin Sidat

**Affiliations:** 1 Department of Community Health, Faculty of Medicine, University Eduardo Mondlane, Av, Salvador Allende no.702, Maputo, Mozambique; 2 Department of International Public Health, Liverpool School of Tropical Medicine, Liverpool, UK; 3 Department of Health, Royal Tropical Institute, Amsterdam, the Netherlands

**Keywords:** Referral system, Community health programs, Community health workers, Enablers and barriers, Primary health care, Mozambique

## Abstract

**Background:**

Effective referral systems from the community to the health care facility are essential to save lives and ensure quality and a continuum of care. The effectiveness of referral systems in Mozambique depends on multiple factors that involve three main stakeholders: clients/community members; community health workers (CHWs); and facility-based health care workers. Each stakeholder is dependent on the other and could form either a barrier or a facilitator of referral within the complex health system of Mozambique.

**Methods:**

This qualitative study, aiming to explore barriers and enablers of referral within the lens of complex adaptive health systems, employed 22 in-depth interviews with CHWs, their supervisors and community leaders and 8 focus group discussion with 63 community members. Interviews were recorded, transcribed and read for identification of themes and sub-themes related to barriers and enablers of client referrals. Data analysis was supported by the use of NVivo (v10). Results were summarized in narratives, reviewed, discussed and adjusted.

**Results:**

All stakeholders acknowledged the centrality of the referral system in a continuum of quality care. CHWs and community members identified similar enablers and barriers to uptake of referral. A major common facilitator was the existence of referral slips to expedite treatment upon reaching the health facility. A common barrier was the failure for referred clients to receive preferential treatment at the facility, despite the presence of a referral slip. Long distances and opportunity and transport costs were presented as barriers to accessibility and affordability of referral services at the health facility level. Supervisors identified barriers related to use of referral data, rather than uptake of referral. Supervisors and CHWs perceived the lack of feedback as a barrier to a functional referral system.

**Conclusions:**

The barriers and enablers of referral systems shape both healthcare system functionality and community perceptions of care. Addressing common barriers to and strengthening the efficiency of referral systems have the potential to improve health at community level. Improved communication and feedback between involved stakeholders – especially strengthening the intermediate role of CHWs – and active community engagement will be key to stimulate better use of referral services and healthcare facilities.

**Electronic supplementary material:**

The online version of this article (10.1186/s12913-019-4076-3) contains supplementary material, which is available to authorized users.

## Background

Community Health Workers (CHWs), known as APEs (Agentes Polivalentes Elementares) in Mozambique, play an important role in increasing access to primary health care (PHC) services, especially for vulnerable and poor communities in low- and middle-income countries [1]. In Mozambique, the majority of CHWs have primary level education and are trained to focus on health promotion and disease prevention, while also providing limited curative care [2]. The CHW training curriculum in Mozambique places considerable emphasis on maternal, newborn and child health care, but also includes topics such as first aid and recognition of common diseases prevalent in their rural communities for timely referral to the nearest PHC facility [2, 3]. Previous studies on the revitalized programme in Mozambique have highlighted the importance of supportive supervision and of the interface role [4–6] that the CHWs play between communities and health systems.

Effective referral systems from the community to the health care facility are essential to save lives and ensure both the continuum and quality of care [7, 8] and can influence CHWs’ performance [9]. There is scarcity of studies focusing on identifying and analyzing the factors and forces contributing to an effective referral process, particularly within community health systems. In a recent systematic review on access to health care, including equitable service provision in rural areas in settings with limited resources, only six studies considered the role of CHWs in referral of clients to a health facility in their analysis [10–15]. These studies revealed that when key factors to promote referral and health facility use are in place, CHWs can play an important role in reducing barriers to accessing healthcare that stem from socio-economic status, language barriers, transportation, and sociocultural factors [16, 17].

Two studies carried out in Bangladesh found a number of factors that promote the uptake of referral services from community to health facility level, including: 1) community engagement through development of action plans involving community and facility stakeholders; 2) creation of community self-help groups to support those from poorer households; 3) household education; 4) community funds or reimbursement schemes; 5) implementation of referral slips; 6) advocacy with local government; 7) CHWs accompanying the patient to the facility; and 8) stronger overall health facility services provision [14, 15]. Evidence from Pakistan and Bangladesh found no improvement in the uptake of facility services where there was limited coordination or linkages between the CHWs and the healthcare facility; when CHWs were not supported or incentivized to facilitate the referral; or when there were no participant support groups in place for clients [12, 14].

In Sub-Saharan Africa (Niger and Malawi), a limited number of studies paid particular attention to CHWs activities to maternal and child health [18, 19]. These examined how referral systems can improve antenatal care, labor and delivery, and postnatal care services at the primary health care level, demonstrating improvement of maternal and child health when CHWs are engaged in those activities [18, 19]. To date, there is no published study available on the role of CHWs in client referrals within the Mozambican context. Moreover, while many studies aimed to measure the proportion of clients referred to the healthcare facility through CHWs and list the reasons for referral [7–25], they failed to explore barriers or enablers for effective referral and whether feedback was given to the referring CHW.

### Mozambican context

In Mozambique, healthcare is mainly provided by publicly implemented national health services. There are clear guidelines for client referral within the national health services, including for the CHW program. CHWs often represent the first point of contact for people in rural areas; clients attended by CHWs in their communities who require further care are referred to primary health care facilities where CHWs’ direct supervisors are based. Each healthcare facility has a specifically defined catchment area, where several CHWs work [2]. It is expected that CHWs are placed within 8-25 km radius from the healthcare facility of reference, thus allowing the CHWs easy access to the healthcare facility and their supervisors [2, 3]. Also, the limited radius aims to facilitate community visits with CHWs supervisors, which allows monthly interactions between CHWs, their supervisors and community members. It is aimed by Mozambican Health authorities that the CHW program increases coverage though the healthcare services provided by CHWs as well as promoting uptake of services at primary health care facilities through the community health referral sub-system [2].

**Fig. 1:**
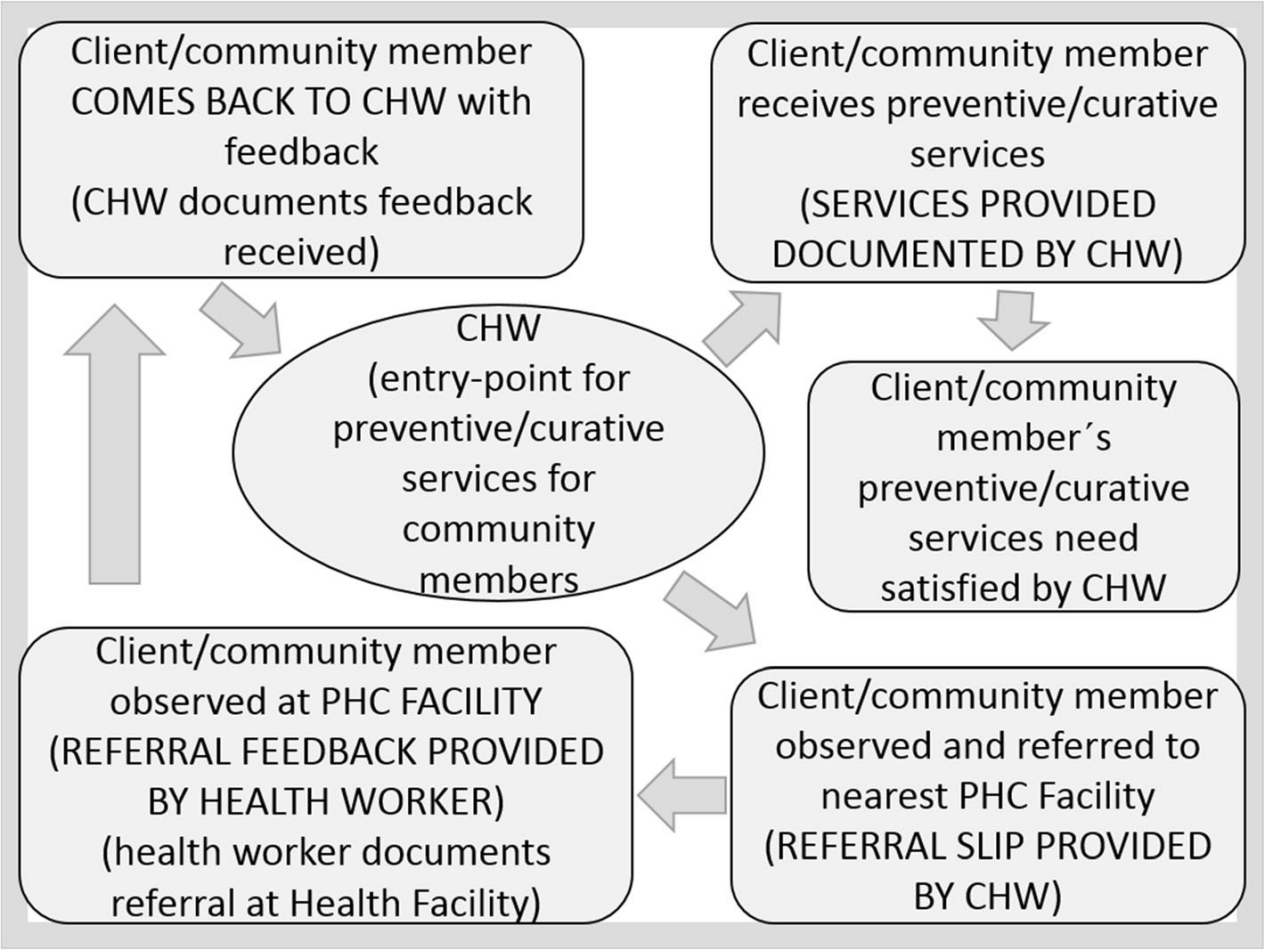
Interaction of key stakeholders in the community-based referral system, Mozambique

The effectiveness of referral systems within community health programs in Mozambique depends on multiple factors that involve clients/community members, CHWs, and facility-based health care workers. Each group is dependent on the other and could form either a barrier, or a facilitating force to uptake of referrals, within the realities of complex adaptive health systems. Thus, our analysis considers the main components of health system strengthening within a system thinking framing, which emphasizes the importance of relationships and the unpredictable behaviours that arise from interactions between system components [26]. Systems thinking recognizes that parts are not disconnected from the whole and that dynamic relationships exist which shape, and are shaped, by the environments in which they are embedded and that network structures represent a broad set of collaborative approaches that are useful for bringing stakeholders together [26]. Figure 1 highlights the interactions within the referral pathway, as developed by the authors, inspired by the Mozambican official guidelines for referral within the CHW program [3].

### REACHOUT/intervention

This study was carried out as part of REACHOUT project, an international consortium representing six low- and middle-income countries in both rural and urban areas across Africa and Asia. The REACHOUT consortium has led research into the implementation of quality improvement activities to strengthen the performance of CHWs and other close-to-community providers in health promotion, disease prevention, and in the provision of curative care at primary health care level. REACHOUT conducted qualitative research across the six countries to identify factors that facilitate or undermine effective, efficient and equitable close-to-community health services provision.

In Mozambique, the initial qualitative research identified the weak referral process as a critical challenge due to its limited functionality, lack of feedback [27] and misunderstanding of the centrality of referral in quality of care [22]. We define referral as having three steps: a written (or verbal) recommendation that a community member seeks services at a health facility; uptake of the service and feedback to the community health worker who made the recommendation. A functional referral system requires two-way communication and coordination between different levels of the system as CHWs play interface role. Thus, functional referral system is assumed to be an appropriate link between health system and the community, by assuring continuity of care and enhancing access to more diverse health care services. Effective referral also contributes to reduction of health care access inequity and better serves the needs of health services for remote rural communities [3]. A systematic review conducted by Kok et al. al found that at the time of the review there was no evidence that functional referral affects CHW performance and we are not aware of papers that have come out since to demonstrate this direct link. Instead this is likely to be an effect mediated through other factors such as supervision, capacity development through feedback and improved communication and coordination with the health system [9].

### The Mozambican REACHOUT team implemented several activities including

- Workshop on referral system involving APEs supervisors from both Districts (sharing of guidelines and tools developed by MoH or MISAU as known in Mozambique; expected roles and responsibilities within referral context for APEs and healthcare workers as per established MISAU standards; importance of referral system in enabling equitable and universal healthcare services access to rural communities in Mozambique);
- Implementation of referral tracking tool developed within the REACHOUT Project to be used at healthcare facility level (adapted from existing MISAU tools);
- Monitoring of referral tracking tool and immediate feedback to improve referral by REACHOUT Project team (at healthcare facility level through APEs supervisor as well as with interaction with APEs directly during field visits).

We reintroduced referral slips, used by CHWs to refer clients from the community to the health facility. The referral slips filled out by CHWs contain client information on the diagnosis, treatment administrated and the reason for referral to the health facility. When the client receives care in the health facility, is expected that the health professionals based in these health facilities will provide feedback to the CHWs on the diagnosis and treatment provided at the health facility. In addition, we trained CHWs supervisors (nurses and health professionals based at the CHWs health facility of reference) in supportive supervision (see Perceived Supervision Scale (PSS) in http://www.perceivedsupervisionscale.com or Additional file 1). One focus of the training was correct referral in order to enhance supervisors’ understanding of the importance of implementing effective referral systems within their rural communities. This was done to improve the scope and quality of health care services provided and community clients’ satisfaction. By comparison, in other districts in Mozambique, CHW supervisors don’t receive training in supportive supervision, referral slips are not available and referrals are done verbally. In this paper we aim to explore the barriers and enablers to effective referral processes within the CHW program in two rural districts of Maputo Province, Mozambique, within the complexity of the health system.

## Methods

We conducted a process evaluation using qualitative methods, performing in-depth interviews (IDIs) and focus group discussions (FGDs) to explore the barriers to and enablers of referral by CHWs to the healthcare facilities and to understand the perspectives of the different stakeholders [28–31] as presented in Fig. 1 [23, 24]. IDIs were carried out with CHWs, their supervisors and community leaders, while FGDs were conducted with community members. Both IDI and FGDs topic guide were developed for this study (see IDIs and FGDs Referral Topics Guide, Additional file 2). Research was conducted in the districts of Manhiça and Moamba of Maputo Province, in the southern part of Mozambique, during April to May 2017, after 4 years of implementation of referral tools.

The interviews with CHWs focused on the CHW program overall, exploring the challenges that CHWs face in their daily activities and what they do to overcome these challenges, particularly with respect to clients’ referral. We also explored the follow-up process after referral – this includes feedback received from the healthcare facilities through referral-slips and/or other modes of communication to understand the overall process of referral. IDIs conducted with CHWs supervisors focused on referral process management including communication and feedback to CHWs, as well as record keeping practices for tracking clients referred to the healthcare facilities. With community leaders the interviews focused on the community perceptions of referral, reasons for (dis) continuing care after being referred to health facilities. Interview topic guides were designed to capture factors that enabled or presented barriers to an effective and fully-functional community health referral sub-system (understood as appropriate and effective links between health system and community with continuous and regular feedback between health facility and CHWs) [3].

The FGDs aimed to explore the perceptions and experiences of community members on referrals made by CHWs. FGD questions investigated community members’ experiences with the referral process; whether or not they followed through with the referral process recommended by the CHW; barriers faced in accessing health care facilities; and experiences and satisfaction with services received following referral. Both IDIs and FGDs were conducted by two experienced qualitative method researchers from REACHOUT Mozambique and were undertaken in Portuguese and when necessary in local languages of Ronga and Xi-Changana requiring translation to Portuguese as they were transcribed. Since the local language is spoken and not written, tools were in Portuguese and their use in the local language required some thinking through and consensus by the team.

### Data analysis

IDIs and FGDs were recorded and transcribed verbatim. Transcripts were read by a team of five researchers for identification of themes and sub-themes related to barriers and facilitators of client referrals by CHWs. It was through this consensus building approach that a coding framework was developed. The analysis process was supported by the use of qualitative analysis software Nvivo (v10) for coding of transcripts. The transcripts were then further analyzed by running queries according to the main themes and sub-themes, while more complex queries looked at sub-groups. Query results were summarized in narratives for each theme and sub-theme, which in turn were reviewed, discussed and adjusted [15].

### Quality assurance and ethics

All interviews were carried out by trained researchers with experience in undertaking qualitative research in community health. IDI and FGD transcripts were randomly checked against digital audio recordings for quality assurance purposes. Informed consent was obtained from all study participants and all collected data remained confidential. To ensure the anonymity of respondents when presenting quotations, district and healthcare facility supervisors (catalogued as CHWs supervisors in Mozambique) are grouped into a single category called “CHWs managers”.

Ethical clearance was obtained from the ethics committee at Liverpool School of Tropical Medicine and the Institutional Bioethics Committee for Health of the Maputo Central Hospital/Faculty of Medicine of University Eduardo Mondlane (reference number CIBSFM&HCM07/2013). After this approval in 2015, an amendment was submitted to add an activity related to the supportive supervision and referral tracking tools. Administrative approval was obtained from the Maputo Provincial Health Directorate and the District Health Directorates of Manhiça and Moamba.

## Results

Twenty-two (22) IDIs were conducted with community leaders, CHWs and their supervisors. A total of eleven (11) CHWs (six female and five male), six (6) supervisors (two female and four male) and five (5) community leaders (four male and one female) participated in IDIs. We conducted eight FGDs with a total of 63 community members (to facilitate free discussion, separate FGDs were held for male (16) and female respondents (47)) (Table 1).

**Table 1:** Sociodemographic characteristics of participants

Informant type		Community leaders	Community members	Health Facility supervisors	Community Health Workers (CHWs)
Data collection method		IDI	FDG	IDI	IDI
Total sample		*n* = 5	*n* = 8	*n* = 6	*n* = 11
Sex	Male	4	16	4	5
	Female	1	47	2	6
Age (years)	18–25	0	13	1	4
	26–35	0	23	4	4
	36–45	2	24	1	3
	≥ 46	3	3	0	0

### Perceptions of the referral system

The value of CHWs health services within referral system is highly recognized by community members and CHWs supervisors. They perceived CHWs as forming an important link between communities and healthcare facilities, and they acknowledged the significance of CHWs in the clients’ referral journey:

*“The referral is very important to follow up the patients. We know that the CHWs cannot treat many diseases; they treat malaria, diarrhea, cough and other simple diseases. The CHWs help us to reach patients in the communities and this can improve our primary health care.”* (Supervisor, female)

CHWs perceived that they are key in linking communities with the healthcare system, and as such they recognized their role in the referral system in order to improve primary health care. CHWs characterized themselves with statements such as “I’m the bridge to link the community to the health system” and “the members of the community acknowledge me as the one who provides health services in the community”, explaining their role in strengthening primary health care and the referral system.

*“I am the one who is responsible for health in the community. It is just me who can provide services to them, when I see that this disease I cannot cure I have to refer, if I don’t do that, who will do it? Helping my community motivates me.” (*CHW, female)

Most supervisors thought that the referral system was well established. They reported the availability of referral slips and guidelines for the CHWs on when, why and how to refer clients. Also, supervisors were aware of the importance of providing feedback to CHWs within the referral and feedback process, so that CHWs understand the client’s diagnosis and treatment, which may help them to more readily identify future diagnoses of the same type.

*“I think that the referral system was well designed. We have referral slips that CHWs use in the communities, and if the person presents the slip he or she is quickly attended. CHWs know what disease they can treat and what not and why. After referral, in the slips we have a space written “clinical comments”, so in this space the healthcare professional who takes care of the referred patients must give comments and the patient should return the slips to the CHWs.”* (Supervisor, male)

### Reasons for referral, according to CHWs and community members

Most CHWs were clear about the types of diseases or health concerns that are beyond their level of competence, including referral of pregnant women for antenatal care and delivery at the healthcare facility by a skilled professional. Similarly, community members also expressed knowledge about why and when clients are referred to health facilities by CHWs. Expressions like, “some diseases he cannot treat”, “he does not have complete training”, or “our nurse does not have all types of medicines” characterize the community perceptions about the reasons why they are referred.

*“When I go to meet our nurse [CHW], usually he gives me the medicine that I want and the correct one. However, sometimes we have diseases that he cannot give the medicine for, other times the medicines are not available. He writes a paper and gives it to us to take to the Manhiça-referral health facility.”* (Community member, female)

Additionally, the CHWs comprehend the referral procedure: giving the clients the referral slips as well as information about the disease diagnosed (if applicable) and the treatment as well as offering them insight into the procedures to follow at the healthcare facility of reference.

*“When I see that this is a serious malaria problem, I send the patient to the hospital using a referral guide. I write something like ‘this patient is sick, is feeling it more’ and then I sign it and then say I will come to your house to see if they [visited] the hospital or not and to see what the health worker wrote.”* (CHW, female)

### CHW stock-outs led to need for referral

It was also found that stock-outs of medicine forced CHWs to refer for diseases such as malaria, diarrhea and other common diseases in the communities. In this situation, CHWs felt uncomfortable and incompetent, because they referred clients with diseases they should be able to handle. This perception of weakness can undermine efforts to improve community health programs and access to primary health care in rural areas. This dilemma of stock-outs and the resulting feelings it triggers was described by one CHW:

*“It is difficult when the CHW has to leave the community to go to the health facility just to take 10 blades, and maybe 25 malaria tests. The community knows that this person can only deal with first aid; the rest is for health facilities. When a patient comes in and you say ‘I don’t have the test or the anti-malarial’, you are creating confusion. So, I send them to health facilities, sometimes it’s very difficult for me, but I have to refer.”* (CHW, female)

### Facilitators of referral

#### Communication between CHWs and supervisors

Continuous communication between CHWs and supervisors was recognized as essential to a strong referral system by both CHWs and supervisors. This was felt to contribute to tracking of clients and to the feedback and motivation of CHWs.

*“It’s important to have communication with our supervisors. For example, my supervisor asked me about how many people I had referred to the health facility a month ago. This can help us to control whether the people referred to the health facility went or not and shows that my supervisor cares about my job.”* (CHW, female)

*“Many people living in the community’s respect and appreciate the CHWs. Our work close to CHWs helps us to get more people from the communities, they link ‘us’ [the health facility] to the communities and this can improve the referral system. As a supervisor I try to have regular communication with the CHWs, but sometimes it is not possible, but we are doing our best.”* (Supervisor, male)

#### Use of referral slips

The existence of referral slips was identified as a facilitating factor for referral by many CHWs and community members. This is related to the norm that, when a client is referred to the healthcare facility and presents the referral slip, they should receive “expedited” treatment, since clients referred by CHWs are not expected to wait in a queue with other clients who have not been referred.

*“We cannot lie to them. When we go to the hospital for referral, with the slip that our ‘nurse’ gives us, they meet us without delay, with no need to stand in the long queue. I even went last month, and it did not take me long.”* (Community member, female)

The referral slips were viewed as confirming that the client was attended by the CHW. As a follow-up tool for healthcare services within the CHW program, the referral slips allow clients to be attended to at the health facility without following normal administrative procedures, such as registration. Thus, these slips were widely described as central to the referral system and to attract clients for continuation of care.

*“The referral slip is a document that the community takes to the health facility. This document helps us as CHWs to refer people without constraint. When you have these slips, you can be attended quickly.”* (CHW, male)

Nevertheless, while there is the perception of referral slips as a facilitating factor, when they are not used for the intended purpose it generates barriers.

#### Pragmatic problem-solving approach

To improve information flow and to support CHW motivation, CHWs, in collaboration with the healthcare facilities they refer to, adopted mechanisms of feedback on the diagnosis and treatment, such as short message services (SMS) and phone calls, however SMS was more used because is less expensive compared to phone calls. These mechanisms emerged to respond to the limited feedback between health providers and CHWs after clients were referred. Envisaged mechanisms for feedback, which require the health provider to write feedback notes on the referral form, were not widely used and/or implemented according to the CHW referral guidelines^1^.

*“The boss [supervisor of the health facility] receives the sick person when I send him there after doing analysis, and he gives the treatment. Afterwards they write on the referral slip about what the patient had and which medicine they gave, for me to know and learn so that when I am receiving the same kind of sick person I already know what can be done. However, people do not always come here after returning from the hospital, so I have to go to their homes anyway.”* (CHW, female)

Many CHWs claimed not to receive feedback from the healthcare facility providers on the referral slips after clients were referred:

*“I don’t know what happened in the hospital if the diagnosis that I made was correct or not. The facility should annotate what they give to the patient so I get to know. With this information, I would be able to know whether the person really went to the health facility.”* (CHW, female)

### Barriers to the referral system

#### CHWs reported three main barriers to referral

1. CHWs observed that some clients did not receive the services and treatment they expected at the healthcare facility after referral, or they did not receive preferential treatment as expected.
2. Some referred clients did not go the health facilities for reasons unknown to CHWs and there were other referred clients of whom CHWs did not know whether they went or not.
3. CHWs felt demotivated when they needed to refer their clients because of their own medicine stock-outs when dealing with a health issue that they are capable of treating.

Barriers number one and three were also identified by a majority of community members although they differed from those identified by supervisors, which focused on challenges with the use of referral data.

#### Treatment at the facility was not as expected

As mentioned above, referred clients expected preferential treatment at healthcare facilities. This did not always happen because some health professionals in health facilities were either not familiar with the guidelines related to CHWs client referral or ignored these guidelines. As a result, many clients became frustrated when these expectations were not met. Frustrations also occurred when CHWs were not able to handout referral slips to referred clients for a number of reasons, including not having enough stock or readily available referral slips with them. Also, as aforementioned, some clients with a referral slip still needed to wait in the queue once they arrived at the healthcare facility, which may have led some CHWs to refer clients without handing out a referral slip. The experiences of community members when they attended healthcare facilities shaped their satisfaction and assessment of the quality of healthcare provided at the facility.

*“We go to health facilities where we receive care, but the problem is that we are treated like other patients who are there. The problem is that our CHWs don’t have the referral document that shows that we are coming from the community to the health facility, because we don’t have a way to be treated in the community. He doesn’t have the document, he just tells you to go the health facilities.”* (Community member, female)

This frustration with the quality of care delivered in the health facilities led some community members to decide to put their faith in God and to seek local sources of treatment, as presented in this narrative:

*“In the hospital they don’t treat us well. I prefer to have treatment with our doctor (CHW). Sometimes they – health facilities’/health providers – just look at you, when you are suffering. The paper – the referral slip – doesn’t work there…. Therefore, some people go to the church or take local medicine.”* (Community member, female)

This challenge was also highlighted by a CHW:

*“We had problems. I have seen cases of my colleagues who have used the referral slip for referral to the facility. ...When the patient arrived there with the slip and presented it, the nurses said ‘you’re sick, and these people who are here are not sick? Join the queue.’ (…) So, when I take a referral slip, it does not help you at all.”* (CHW, male)

#### Patient failure to take up referral

The distance to reach the healthcare facilities, lack of access and costs of transport were presented as major barriers by community members and CHWs that prevent clients from following the referral pathway recommended by their CHW. Other barriers were the costs associated with healthcare received, such as for medicines, laboratory tests, or for unjustifiable payments requested by healthcare professionals (corruption practices). The communities prefer services close to where they live, provided by CHWs or other providers, such as traditional healers. Furthermore, most community members perceived CHWs services to be of better quality if compared to the services delivered in the health facilities, and found CHWs manner friendlier and more empathetic than those provided at the health service level/hospital and other health facilities:

*“Sometimes you don’t have money to go to the hospital. Look, from here to Moamba healthcare facility is far, you can walk 6 hours, to find transport is difficult, sometimes we have to wait for a lift, if the vehicle doesn’t come you can’t go there. How can you do what they tell you to do? We ask to have more medicines here and increase the knowledge of the CHWs. He treats us very well and better than there – at healthcare facilities.”* (Community member, male).

#### Barriers identified by CHWs supervisors

In contrast to the common barriers identified by CHWs and community members, supervisors tended to identify barriers to the use of referral data itself, rather than barriers to the uptake of referral by clients. Barriers to the referral system identified by supervisors can be summarized into three categories: a lack of culture of feedback among supervisors and CHWs; high workload of supervisors; and the underutilization of information generated in the referral slip by supervisors. Although identified by CHWs, supervisors indicated that the lack of feedback was widespread and occurs throughout levels of health system in Mozambique not just at community level, as suggested by one of the supervisors:

*“The experience that I have in the health system in Mozambique allows me to say that there is no culture of feedback between health professionals. We have the documents that recommend you to do that, but they are not used. For example, if you are referred to Maputo Central Hospital – a major hospital of Mozambique – the doctor who receives you should send a document back to the hospital that referred you, but this doesn’t happen. I think that the same is happening in primary health care.”* (Supervisor, male)

Regarding the second barrier – workload – supervisors argued that the time that they take to conduct daily tasks, with enormous numbers of patients they need to attend per day does not give them sufficient time to pay attention to the CHWs and community members’ referral challenges.

*“The supervisor of the CHW usually is responsible to monitor the CHW’s work. He has many activities to do, sometimes it is not easy for him to follow all procedures and have attention for the CHW’s activities. Another thing is that the data provided by CHWs in referral and other activities are not used. It is difficult to progress in this way.”* (Supervisor, male)

Third, the underutilization of information generated in the referral system by the supervisors undermined the effort to fortify the referral system and primary health care in general. Information describing the challenges encountered in the referral system process was not used by the supervisors to identify the weaknesses and strengths of health service delivery. Also, the number of clients referred by CHWs to the health facility was not well documented, leading to a lack of information on trends of clients referred by the CHW and the main reasons for referral. Record keeping at healthcare facility level was problematic and this challenge goes beyond the CHW program.

## Discussion

Our findings reveal that all participants acknowledged the importance of the intermediate role of CHWs in referral and its contribution to the continuum of care and to enhancing quality of care. CHWs and community members identified similar facilitators and barriers to uptake of referral. A major common facilitator was the existence of referral slips to expedite treatment upon reaching the health facility. At the same time, a major common barrier was the failure to receive preferential treatment at the facility, despite the presence of a referral slip. Additionally, long distances and financial costs created barriers to the accessibility and affordability of referral services at the health facility level. Supervisors identified barriers regarding the use of referral data, rather than uptake of referral. Both supervisors and CHWs saw the lack of feedback as a barrier to a functional referral system.

In a previous study carried out by the REACHOUT consortium in Ethiopia, Kenya, Malawi, and Mozambique, the referral system at times lacked effective reporting procedures and feedback systems, which hindered communication between the CHW, the health facility and the clients/ community [4–6, 32, 33]. Limited feedback systems were felt to influence CHW’s relationships with the community, leading communities to have limited confidence in the referral services and in the CHWs who link the community and health facility [6, 34].

Our findings confirm a number of the facilitating factors also identified through previous studies, including: use of referral slips; community engagement to understand reasons for referral and the presence of a strong relationship between the CHW and their supervisor [13]. A number of barriers were also identified, including failure to receive preferential treatment at the facility. This is in line with previous studies which found that mixed messages or limited coordination between CHWs and health facilities reduces effectiveness of feedback and referral [8, 11]. Additionally, failure to address barriers to access, including affordability, continues to hinder clients from taking up CHW initiated referral [6]. The creation of community self-help groups or introduction of community funds or reimbursement for referral related costs, which could possibly be facilitated by CHWs, may go a long way towards overcoming these barriers and increasing uptake of services at the health facility level [11, 12], also strengthening community engagement and ownership of close to community health services. Community engagement approaches that achieve shared leadership with the community have been shown to be successful at improving linkage between health system and community [35]. Crucially, adopting a community action cycle approach, which incorporates learning, action and dialogue within the community may prove most effective [35].

As in any health system is important to ensure the “hardware” elements of CHW programs that allow the functioning of referral systems are in place. Availability of medicines and other supplies (e.g. tests) at community level would allow CHWs to treat what they can at the community level – this will relieve the burden on the referral system and the health facilities. The structure of and (reporting) tools used in the referral system, or “hardware” elements, however, can only lead to effective care when the “software elements”, in particular trusting relationships between the three different groups of stakeholders, are present [6]. As presented in Fig. 1, a well-functioning referral system within community health programs and complex and adaptive health system of Mozambique have many nuances and are configured by many elements involving clients/community members, CHWs, and health care workers. These groups are interdependent and can constitute either a barrier or a facilitating factor within the Mozambican health system. Our study shows that there are sometimes limited interactions between these three groups, which has a negative impact on the referral, trust in the health system and ultimately could lead to poor health outcomes. The lack of trust is demonstrated by the frustrations felt by community members when they decide to not seek health care at all, risking adverse effects of treatments outside of the formal health system or preferring to place their faith in God to improve their health conditions. This situation aggravates the risk and vulnerability of community members. Gilson, as argued on the centrality of communication and management of relationships to give a value and meaning of health services to the community or persons and to build a trust [36] and in our research trust is one of the components that emerge as barriers of referral system within CHW program in Mozambique.

A well-functioning referral system may serve as a motivating factor for CHWs by guaranteeing a continuum of care for the client, which can in turn can boost the relationship of CHWs with the community. Furthermore, the referral process has the potential for improving CHWs’ skills and knowledge, by understanding clients’ diagnosis and treatment once they return to the community. In complex and adaptive health systems, strong and continuous linkages and interactions between clients/community members, CHWs, and health care workers can improve referral, strengthen the health system and contribute to better health outcomes.

### Study limitations

Our study highlights the challenges of primary health care provision and CHW program implementation in Mozambique but has several limitations. First, a lack of literature on referral systems in primary health care, especially from a community to health facilities, undermines comparative analysis of the dynamic of the referral system in limited resource settings, particularly in Mozambique. Poor documentation of the referral process and the number of clients referred by CHWs to health facilities limited our ability to understand trends within the referral system and to measure outcomes of CHW referral. This study was developed in two districts of Maputo Province in southern Mozambique, so findings might not be generalizable to other geographic regions of the country. Lastly, we did not particularly explore community-based factors influencing referral, such as self-help groups and village level saving.

## Conclusions

The barriers and facilitators of referral shape both healthcare system functionality and community perceptions of care and are important to address to improve health at community level. To enhance access to, and the quality of, primary health care in Mozambique, particularly in rural areas, a fully-functional and effective referral system is needed. Improvements in referral can be achieved by strengthening communication and feedback between CHWs, supervisors and health professionals based in the health facilities. This will help the CHWs and their supervisors to better understand the barriers patients face in going to the healthcare facilities they have been referred to and collaboratively help community members to overcome them and improve the continuum of care at community level. Active community engagement and feedback can stimulate better use of healthcare facilities and critical services provided by CHWs and health authorities at community level.

## Additional files

Additional file 1: REACHOUT Supportive Supervision Training Manual. (PDF 20161 kb)

Additional file 2: Referral In-depth Interview Topic Guide 2. (DOCX 15 kb)

## Abbreviations

APEs: Agentes Polivalentes Elementares
CHWs: Community Health Workers
FGDs: Focus Group Discussions
IDIs: In-depth Interviews
PHC: Primary Health Care

## Footnotes

1 See figure 1 for more details.
