# Supplementary material for: Strengthening referral systems in community health programs: a qualitative study in two rural districts of Maputo Province, Mozambique
Source: BMC Health Serv Res. 2019 Apr 29;19:263. doi: 10.1186/s12913-019-4076-3 (PMC6489304; doi:10.1186/s12913-019-4076-3)
Supplement: Supplementary file 1 — REACHOUT Supportive Supervision Training Manual. (PDF 20161 kb) [file 12913_2019_4076_MOESM1_ESM.pdf]

SUPPORTIVE SUPERVISION TRAINING MANUAL FOR COMMUNITY HEALTH EXTENSION WORKERS (CHEWs)

Trainers Manual

Authors: Ndindi Mutisya, Olivia Tulloch, Maryline Mireku

lvct Training Institute
CENTRE FOR PROFESSIONAL GROWTH IN HEALTH

Reachout
Linking Communities & Health Systems

Acknowledgements

We would like to thank the European Union for their financing and support. This document reflects only the authors' views and the European Union is not liable for any use that may be made of the information contained therein'.

We would like to acknowledge the training team at LVCT health for their role in developing and piloting this curriculum.

We would like to thank the support given by Dr. Lilian Otiso (LVCT Health) and Korrie de Koning (Royal Tropical Institute, Netherlands)

Contents

Acknowledgements........2

Course syllabus........5

Module 1: Concept of Supervision........9

Session 1.1: Definition of Traditional and Supportive Supervision........9

Session 1.2: Qualities of a Good Supervisor........10

Session 1.3: Functions and Tasks of Supervision........13

Module 2: Skills required by a supervisor........19

Session 2.1: Effective Communication Skills........19

Session 2.2: Group Facilitation Skills........23

Session 2.3: Problem solving skills........29

Session 2.4: Advocacy skills........35

Module 3: Process of supervision........46

Session 3.1: Introduction to the components of the supervision framework........46

Module 4: Community engagement........61

Session 4.1: What is Community Engagement........61

Session 4.2: Role of Community Engagement in Supervision of CHWs........62

Session 4.3: How CHEWs engage Communities in Community Health Services........63

Session 4.4: Community Dialogue Days........64

Session 4.5: Community Dialogue Can Translate the Problem to Become a Community Concern........67

Module 5: Monitoring and Evaluation in Supervision........69

Module 6: Ethical considerations in supervision........72

Session 6.1: Code of Ethics in Supervision........72

References........74

Part 1

COURSE SYLLABUS

Course syllabus

Course description

This curriculum is for training Community Health Extension Workers (CHEWs) in carrying out supportive supervision of Community Health Workers (CHWs).
This course is designed to equip participants with knowledge, skills and attitudes to effectively support CHWs with an aim of improving quality of service delivery at household level.

General course objectives

At the completion of this course, the participants shall be able to;

- Describe concepts of support supervision
- Demonstrate the skills required by a supervisor
- Understand the process of supervision
- Explain networking, partnership & community engagement
- Discuss ethical considerations in supervision
- Discuss monitoring and evaluation in supervision

Training methodology

The training methodology employed in this training is experiential, participatory and didactic to include:

- Role plays
- Group work and discussions
- Brainstorming
- Case scenarios
- Mini-lectures

Target group

This curriculum primarily targets the Community Health Extension Workers working as supervisors of Community Health Workers (CHWs) under the Community Health Strategy in Kenya.

Course duration

This course is designed to take duration of five (5) days.

Evaluation methods:

Pre and post course assessment
Training course evaluation
Observed practice

Certification

This course is designed to develop competency for implementing support supervision. Competency is attained through successful completion of **all course activities** including;

- Attending at least 90% of the class sessions
- Implementation of the activities on the work plans that will be developed at the end of the course.

<table>
<tr><th>TIME</th><th>MONDAY</th><th>TUESDAY</th><th>WEDNESDAY</th><th>THURSDAY</th><th>FRIDAY</th></tr>
<tr><td>8:00 - 10.30</td><td>Welcome and introduction
Expectations of group
Course goals and objectives
Group norms
Pre course assessment</td><td>Recap

Module 2: Skills required by a supervisor
- Communication skills
o Questioning

- Group facilitation skills
- Group dynamics (animals)</td><td>Recap
o Advocacy skills</td><td>Recap
Practice</td><td>Recap
Module 4: Community engagement
- Definitions of the terms: community engagement
- Process of community engagement
Community dialogue and the steps for community dialogue</td></tr>
<tr><td>10:30 - 11:00</td><td colspan="5">TEA BREAK</td></tr>
<tr><td>11:00 – 1:00</td><td>Module 1: Concept of supervision
- Definition of traditional supervision and supportive supervision
- Qualities of a good supervisor</td><td>- Group facilitation skills (Continued)
o Johari's Window
o Group development stages
o Group facilitation skills including the group focusing skill

- Problem-solving skills</td><td>Module 3: Process of supervision
- Supervision framework
o Supervision components
o 5 Stages in group supervision
o Quality management in supervision</td><td>Practice</td><td>Module 5: Monitoring and evaluation in supervision
- Documentation by CHEWs
- Utilisation of data tools (Process of feedback and dissemination)

Module 6: Ethical considerations in supervision</td></tr>
<tr><td>1:00 – 2:00</td><td colspan="5">LUNCH BREAK</td></tr>
</table>

**2:00 – 4:30**	**Module 1: Concept of supervision**<br>- Functions of supervision<br>- Tasks of a supervisor<br><br>**Module 2: Skills required by a supervisor**<br>Communication skills<br>- Attending skills<br>- Active listening	- Problem-solving skills (continued)	***Module 3: Process of supervision***<br>- Methods of supervising<br>○	*Practice*<br><br>**Wrap up practice and peer evaluation**	**Way forward/ action plan**<br>**Post course evaluation**<br>**Closing**
**5: 00 PM**	**TEA BREAK**				

Module 1: Concept of Supervision

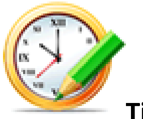

Time: 4hrs 30mins

By end of this module, the participants will be able to:

1. Differentiate traditional supervision and supportive supervision
2. Describe the qualities of a good supervisor
3. Discuss the functions of supervision-educative, supportive and administrative
4. Outline the roles/tasks of a good supervisor

Session 1.1: Definition of Traditional and Supportive Supervision

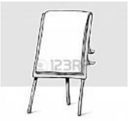

Activity one: Brainstorming definitions (40 minutes)

1. Facilitator to ask the participants to brainstorm how the participants define the following terms relation to community health service providers - **traditional supervision and supportive supervision**
2. Write the responses given by the participants on a flip chart
3. Fill in the gaps or give the right definitions using the notes below

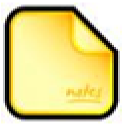

Facilitator's notes

Traditional supervision

Traditional supervision is superficial. It only looks at the face value of the problem and not the root cause. It is often punitive, fault-finding and critical and therefore does not offer solution. It focuses on individuals rather than processes therefore do not endeavor to strengthen systems and processes that may cause disconnect between current outputs of health care to the expected standards. Traditional supervision emphasizes the past rather than the future. Further it's not continuous but

it's rather intermittent and usually it creates resentment, suspicion, disintegration between team members rather than focusing on strengthening team work for quality service delivery.

Supportive supervision

Supportive supervision can be defined as 'a process of guiding, monitoring and coaching workers to promote compliance with standards of practice and assure the delivery of quality care services. The supervisory process permits supervisors and supervisees the opportunity to work as a team to meet common goals and objectives. It is about 'empowerment and not control, emphasizing building confidence and self-esteem through supportive feedback'. It is facilitated through an encouraging and respectful relationship with community health service provider. It sets expectations, monitors and assesses performance, identifies problems and opportunities in which the supervisor remains an intermediary promoting collaboration in problem solving and linking to external resources. The concept of supportive supervision is to place within the health system individuals whose purpose is to coordinate the aspects of the health system which support community health service providers in service delivery. A supportive supervisor's job is to identify everything that his/her community health service providers need to succeed – including continued training, supply of medicines, easy mobility, emotional support etc. and ensure that these supports are in place. Good quality supervision is crucial for community health service providers to remain motivated and active in their jobs and to feel valued in their work. Community health service providers always feel a 'value-add' for participating in a supervision exercise, and not come to fear or avoid it. It should an opportunity to share their concerns, help them to overcome the challenges they have experienced in their work, and to learn more about the work that they are carrying out through the knowledge sharing and coaching of the supervisor. For many, supervision also means 'line-management' and therefore CHWs may feel reluctant to report the difficulties they have. This attitude will limit the effectiveness of the supervision in improving work quality and eventually lead to supervisees becoming demoralised. Creating and *open dialogue and a mentoring relationship* will be most effective in helping them to report, identify and resolve the problems they have and will lead to genuine improvements competencies improvement over time.

Session 1.2: Qualities of a Good Supervisor

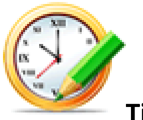

Time: 1hr

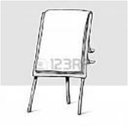

Activity two: Characteristics of a supervisor (20 minutes)

In advance of the training the facilitator writes the characteristics in the table below on cue cards or A4 paper, they should be big enough to display on the wall.

The exercise aims to identify and demonstrate the qualities and characteristics of good supervisors. The characteristics may not always come naturally and so supervisors may need to cultivate the characteristics.

SUPERVISOR		
Develop supervisees' capacity	Motivate	Empower
Provide Feedback	Reinforce	Support
Guide	Set goals	Influence
Create or maintain a working relationship	Advise	Inspire
Ensure tasks are completed	Correct	Model accountability
Explain What to Do	Tell what NOT to do	Confront
Help think through personal problems	Listen	Direct
Discipline	Discuss	Mentor
Develop problem-solving skills	Reflect	Advocate for your needs
Maintain clear performance standards	Discuss	Observe
Provide Perspective	Share Expectations	Praise

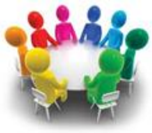

Activity three: Group discussions (40 minutes)

- Divide the group into pairs and give each pair several of the pieces of paper with the qualities written on them.
- Ask the group to consider the qualities and discuss the following questions: Are they important? Are they useful? Ask them to come up with an example situation that illustrates the point. It could be a real situation from own experience or imagined situation
- Ask the members to share examples with the larger team (try to keep these presentations as brief as possible)
- Ask the participants to write down the qualities that they think will be easiest for them to perform and those that will be difficult.
- You will display the pieces of paper on the wall, with an area of the wall for Easy and an area of the wall for the Difficult. First discuss each quality with the whole group and reach consensus about which ones are difficult or easy.
- You should review the results. Do the participants all choose different things? Are their opinions mostly all the same? Is there one area of leadership that stands out as seeming most difficult for many of the participants? Spend some time discussing with the participants about ways that they can develop these skills, referring to the notes below about the qualities of a good supervisor. Inform the group that by the end of the training it should be possible to move all of the characteristics away from Difficult.

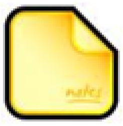

Facilitator's notes

The attitudes and relationships that develop between the community health service provider and supervisor are just as important as the day-to-day duties a supervisor will have. Supervisors provide the example for teaching and learning that community health service providers follow when they are

in their communities. If the supervisor bosses and “talks down” to community health service providers, the community health service providers are more likely to “talk down” to community members. However, if the supervisor treats them as equals, notices their strengths and builds on them, they will be more likely to work the same way with people in their communities.

Qualities of a good supervisor

- Good listener
- More knowledgeable in technical issues than the community health service providers (It is ok for the supervisor not to know everything, but they can enquire)
- Resourceful e.g. being able to make things happen when confronted by obstacles. This may be referred as having problem solving skills but it’s also about being innovative and thinking ‘out of the box’-being creative
- Have good communication skills
- Be a role model-be respect, able to give community health service providers space to air their issues, able to show interest by the way they listen, acknowledging the strength of others
- Able to observe confidentiality
- Being respectful – it’s more than being courteous and polite. Being able to treat people as individuals, acknowledging their individual needs and aspirations
- Supportive e.g. being attentive to the community health service providers personal and professional needs; absence of a superiority complex; assistance with challenging aspects of the community health service providers work, being flexible like having flexible working hours
- Be a mentor and coach the community health service providers for good performance
- Knowing and being able to use the community health service provider’s strengths
- Allowing the community health service providers to manage their own time and workload
- Fostering a relationship between the community health service provider and the clinic and hospital, to help integrate them into the local health system
- Ability to motivate community health service providers to continue to improve their skills
- Taking the time to give frequent, constructive feedback on the community health service providers’ performance

It may require that you as the supervisor start to think differently about what it means to be ‘in-charge’. Though supportive supervision will take some practice, in the end we believe it will produce better results and ultimately help save lives.

Barriers of being an effective supervisor

The barriers just identified are real, not imagined. Don’t feel bad that you can’t be an *ideal* supervisor all the time—nobody can! An effective supervisor knows their own strengths and weaknesses and learns to use those strengths and weaknesses wisely in day-to-day interactions with staff.

The first step to becoming an effective supervisor is to know one’s own strengths and weaknesses. Since you’ve identified what you think an ideal supervisor looks like, and those things that happen

that get in the way of being an ideal supervisor, spend a few moments thinking about what you need to overcome your barriers and improve your skills

Supervisors have a powerful impact on workers' lives. A worker's relationship with his or her supervisor is often the most influential factor in whether the worker feels valued and respected at work. Not surprisingly, feeling valued and respected is one of the biggest factors affecting a worker's decision to stay on the job or quit.

Session 1.3: Functions and Tasks of Supervision

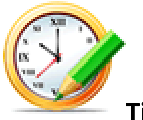

Time: 2hrs

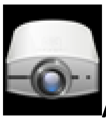

Activity four: Functions and tasks of a supervisor (60 minutes)

1. Give a brief presentation on the functions and tasks of a supervisor (*see facilitators notes below which can be presented as a PowerPoint presentation that accompanies this curriculum)*
2. Divide the participants into three groups, and allocate each group a function: supportive, educative or administrative. Ask them to give examples of the tasks they have in relation to the function they have been allocated.
3. The groups share with the larger group their responses.
4. Facilitator fills in the gaps with the correct information as guided by the facilitator's notes below.

Questions for discussion

In your experiences of being supervised, which experiences did you find helpful and which did you find most unhelpful?

In your experience as supervisors, what roles have played? What experiences have you found helpful and what have you found most unhelpful?

Does it sometimes happen that supervisors only worry about the work performance and do not show support or understanding?

Is it possible to be concerned about performance but in a supportive way?

Supervisors often focus or only have time for the administrative tasks. Highlight the importance of supportive supervision.

Some examples of supportive supervision are: Give information/advice: supervisors offer options / psycho-social support e.g. build rapport so the supervisee can turn to supervisor for advice or a listening ear, and opportunities to refer to counselling / accompany on normal activities if they want assistance on issues that have been identified/ Empower them to form CBOs to improve finance / Visit at home if are in strife (e.g. family death) / Action-lining: Bonding session where the supervisees come together to air their problems, and community reciprocation / Build a relationship / be approachable.

Facilitator’s notes

SLIDE PRESENTATION on Functions and tasks of supervision

- **Functions of supervision**

Support supervision has three main functions;

1. Educative (formative)— professional development of the supervisee
2. Supportive (restorative)— welfare of the supervisee
3. Administrative (normative)—quality assurance

1. Educative (formative)

In supervision, knowledge and information (theory base), personal development and skills training is carried out. Supervision is also of learning by doing, allowing supervisees to reflect on their work with in the presence of an experienced person who enables that reflection. Supervisors inevitably fill in gaps in knowledge, increase skills, make practical what was only abstract knowledge but what must now become working knowledge. A link is established between theory and practice. This function has been called the ‘formative’ function of supervision.

Supervision is directive in the behavioural approaches; in the humanistic it is more informal. The facilitative role of the supervisor enables learning in the interaction.

2. Supportive (restorative)

The supportive role of supervision is emphasized more in the person-centred approach. It involves offering supervisees a forum where they are encouraged to look at their own issues and ask for or be given the encouragement they need to explore their way of working with clients. It is this function that provides the ‘containment’ side of supervision. Supporting supervisees as they struggle to work, as they deal with other’s or the community members difficulties, as they engage emotionally with community members, takes place throughout all aspects of supervision. This function of supervision has been called the ‘restorative’ function.

3. Administrative (normative)

The administrative function has an eye on all aspects of the work that contain accountability and responsibility of the supervisee and the welfare of the client.

Called the ‘normative’ function of supervision, it pays heed to the ethical and professional aspects of client work. It enables supervisees to monitor their own work as professionals.

It is here that supervisors become advocates, making sure that quality service is rendered and that ethical and professional dimensions are maintained at a high level.

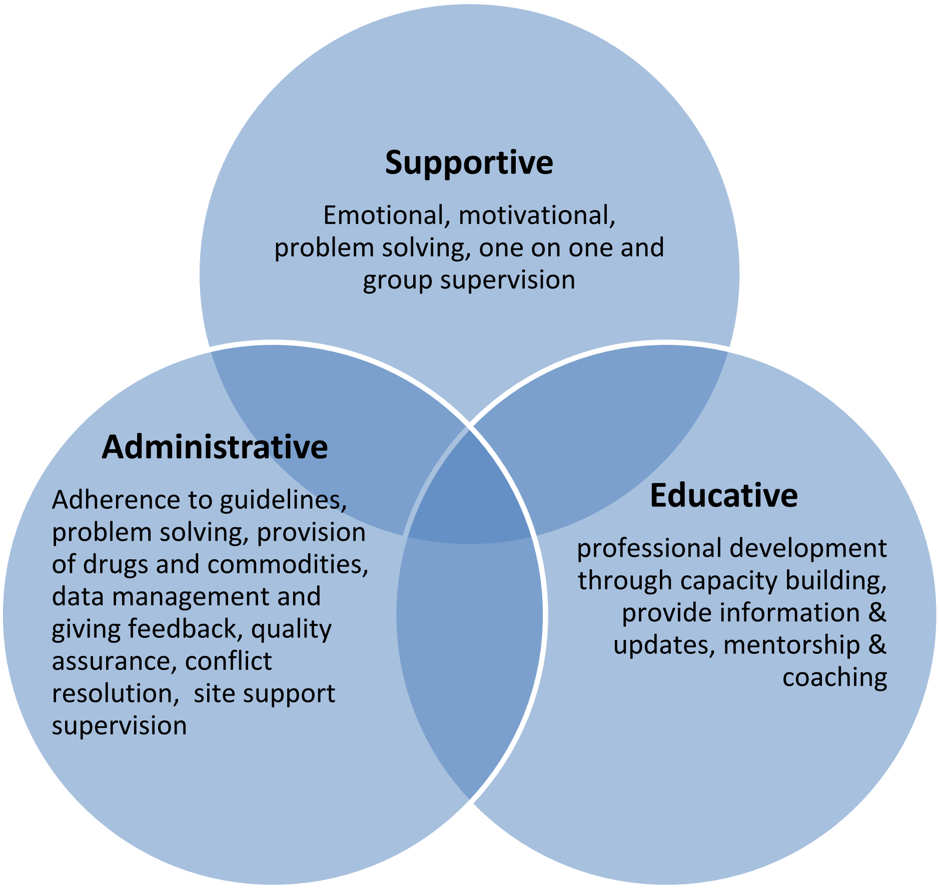

Questions relating to this diagram: *Looking at the pie chart, which is the one that is easiest to do. The administrative? Well that is traditional supervision, it is very easy not to attend to the supportive side of supervision, because it is the difficult side. The educative side? What do you think about that? Do you think they already know what they are supposed to do?*

Supervision as the backbone of a functioning community health service provider system

In a nutshell and in the context of supervision within the community health service provider programme, supervision contributes to:

**Continuing training:**	The mentorship and capacity building aspect of supportive supervision contributes to the up-skilling of the community health service provider
**Equipment and supplies**	Typically assessed through supervision visits
**Individual performance evaluation**	The basis of which comes from the supervision reports and data submitted over the course of the year
**Opportunity for advancement**	Supervision identifies good performance amongst community health service providers and therefore those which merit access to additional opportunities and skills based training can be identified through supervision
**Documentation and information management**	Supervision is typically the point of entry of data into the system – collection, collation, analysis, reporting and dissemination.

**Linkages to the health systems**	Supervision is typically the key way in which community health service providers are linked to the health facility staff. Supervision means building a mentoring relationship between them and individual health providers in the facility, builds a sense of accountability by the facility for the community health service providers providing services in the community.
**Community involvement**	Successful engagement of the community in the recognition of the community health service providers work may particularly involve being able to feed into the supervision processes.

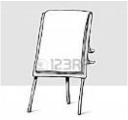

Activity five: Tasks of a supervisor (60 minutes)

Ask the participants if they know what the tasks of supervision tasks are.

There are seven tasks of supportive supervision as outlined below (facilitator's notes). The may not be sure what the tasks are, and you may need to give them the headings for the following activity.

Write the following headings on to flip charts. Each flip chart should have only one heading on it.

1. Relationship between the supervisor and the community health service provider supervision
2. Monitoring the professional and ethical aspects of supervision
3. The supportive task of supervision
4. The teaching task of supervision
5. The evaluation task of supervision
6. Consultation task of supervision
7. The administrative task of supervision

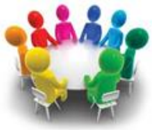

1. Divide the participants into small groups. Depending on the number of participants give each group one or two flip charts. Ask them to discuss how they understand the tasks. For example, Task 1 on relationships, discuss: how you first make contact with a supervisee. What is your first meeting like, what do you do? How did you meet them?
2. Ask each group to feedback their discussion briefly.
3. After each task ask the rest of the group to contribute their ideas.
4. Fill in the gaps according to the notes below.

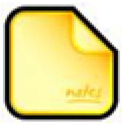

Facilitator's notes:

Tasks of supervision

1. Relationship between the supervisor and the community health service provider

- There is little agreement on the 'kind' of relationship involved.
- The relationship changes as supervision progresses.
- Supervisors feel able to combine other roles with supervisees.
- There is an element of power within supervision.
- The supervision relationship is characterized by choice: self-disclosure, transference, counter-transference and contract for example self-disclosure of the supervisor and the supervisee – how much personal issues do people share? Is it appropriate for supervisors to share their own issues?
- Transference and counter-transference for example where the community health service provider may become dependent, or the supervisor may project issues or displace or where the supervisor sees the other as helpless and takes responsibility rather than helping them know how to solve the problem. As a supervisor you are not supposed to take over a job of the other but to give guidance which is not the case- how do you do that without becoming manipulative.

2. Monitoring the professional and ethical aspects of supervision

- Monitoring the professional and ethical aspects is seen as an essential task of supervision.
- There are different opinions on how to monitor.
- Supervisors need to assure themselves that their supervisees are working ethically.
- Some issues emerge around this for supervisors.
- Supervisors have worked out strategies for dealing with supervisees when they are anxious about the ethical or professional side of their work.
- Supervisors have several options when there is serious doubt about a supervisee.

3**. The supportive task of supervision**

- Supervisors expect personal issues to arise from supervision.
- Supervisors are concerned that the supervisee's personal issues might interfere with work.
- Supervision deals with personal issues as they emerge from work but not personal issues per se.
- Some supervisors work with personal issues only when they throw light on work with client.
- Some supervisors give limited space to deal with personal issues.
- Some supervisors demand action if they are worried about client work.

4. The teaching task of supervision

- Teaching is an essential task of supervision.
- Teaching is individualized within supervision.
- Teaching within supervision is more informal than formal.
- Modeling is seen as an important teaching method.
- Teaching recedes as the supervisee becomes more experienced.
- Teaching methods are used within supervision mostly determined by the supervisor.
- Some supervisors react negatively to formal teaching in supervision.
- Some teaching methods are used such as demonstration, role play, taping etc

•

5. The <u>evaluation</u> task of supervision

- Evaluation is seen as a key task within supervision.
- Ongoing feedback evaluation ought to be built into the supervisory contract.
- Evaluation is the responsibility of the supervisors; it inevitably affects supervisory relations.
- In evaluation the power issues are very clear.
- Supervisors tend to give methods of formal evaluation.
- There are different stances on the use of taped material for evaluation purposes.

6. <u>Consultation</u> task of supervision

- The consultation task is described in various ways by supervisors.
- There is different emphasis on different systems.
- Supervisors want to know what is happening to clients.
- Parallel process must be used with caution.
- Developmental stages are connected to the consultation task.
- The consultation task is widely seen as the underlying key task of supervision.

Includes consulting with management, partners, donors, and even volunteers, what do *they* want changed? Learning from each other, this is the situation in my unit and the problem stems from beyond the unit, so need to consult for others, and ask advice.

7. The <u>administrative</u> task of supervision

- Provide support to community health service providers around logistical challenges (such as transportation) and difficulties with service delivery such as commodity supply. This may also include challenges that occur during household visits.
- Conduct spot-checks to ensure that community health service providers have visited and are providing all required services to the community.
- Accompany individual community health service providers on household visits to assess performance. Look for areas where community health service providers are strong, and where they are weak. Coordinate with central health management to provide incentives for strong performance OR further assessment, refresher trainings, and mentorship for poor performance.
- Improve cooperation between the community health service providers and the health facilities by being a link between them.
- Compile reports
- Provide mentorship to the community health service providers through on-the-job training
- Meet with individual community health service providers to discuss performance and provide constructive feedback and one-on-one
- Ensure quality assurance in service delivery

Module 2: Skills required by a supervisor

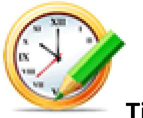

Time: 7hrs 20 minutes

By end of this module, the participants will be able to:

1. Define effective communication skills including attending skills, active listening and questioning
2. Demonstrate ability to effectively undertake group facilitation
3. Demonstrate problem solving skills
4. Demonstrate advocacy skills

Session 2.1: Effective Communication Skills

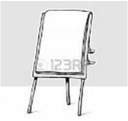

Activity one: Exercise-Broken Telephone (25 minutes)

- Ideally, this activity works with groups of about six to eight people.
- Ask the group to form a line or a circle
- Starting with the first person in the line or anyone in the circle, whisper a one-sentence message in that person's ear (loudly for only that person to hear).
- Ask that person to repeat what s/he just heard into the ear of the person next to or behind her/him and repeat the process until the message is passed to the last person.
- Ask the last person to repeat what s/he just heard into the ear of the person next to/behind her/him.
- Ask the last person to state what was said and check if it was accurate to the message that was given to the first person
- Facilitator then asks the group to describe their observations and reactions to the exercise
- Ask the group why it is important to know that what is said is not always the same as what is heard
- Ask the group why listening is very important for strong communication to take place
    - What did they learn through this exercise that they could apply to their jobs as supervisors?

Activity two: Attending skills exercise

Exercise: Role play

- Divide the participants into groups of 3; ask one person to play the role of a supervisor, the other a community health service provider and the other an observer. These roles can be alternated
- Ask the community health service provider to share a story with the supervisor and the supervisor to act as if he/she is not listening e.g. fidgets on the seat, looks around or stares blankly.
- Ask the group to swap roles, this time the supervisor and the community health service provider to sit back to back as the community health service provider shares the story
- In the last, role play have the supervisor practice the attending skill (SOLER) and the community health service provider shares a story
- In each of these role plays, the observer should take notes of the community health service provider's and supervisor's behaviour
- After each role play, ask the supervisor to repeat what he/she heard as the community health service provider was sharing and then ask the community health service provider how he/she felt
- Then ask the observer to give constructive feedback on the session. Give examples of ways that the supervisor showed good/bad listening skills

This role play also helps the group practice on giving constructive feedback to each other

These two exercises provide the participants with an opportunity to practice communication and listening skills. Active listening – This is the ability to hear well and recall accurately all the verbal information presented.

- Listening involves more than hearing words
- Listening involves attempting to grasp emotions that are often veiled behind the spoken word
- It is the active process of paying undivided attention to what the client is saying and what they are not saying

Facilitator’s notes

Attending skill – This is being fully available for your client.

- Socially- politeness, kindness
- Physically – body posture, being available mentally

SOLER

This acronym stands for:

Sit squarely- to communicate presence and availability

Open posture- to signify that you are open to the client and to what the client is saying. This is seen by others as a non-defensive posture

Lean forward (slightly) - towards the client is a natural sign of involvement

Eye contact- communicating your presence, interest and that you want to hear what the other person wants to say**.** (This should not be confused with starring)

Relax- Relax and remain natural when doing all the above. It means becoming comfortable with your body and the situation**.**

The **Purpose** of SOLER is to demonstrate interest and attention by using body language

Activity three: Effective questioning skill (30 minutes)

Guides to questioning techniques often recommend the 5WH approach. This stands for: What? Why? When? Where? Who? How?

This is a useful starting point for thinking about the sort of questions you might ask. Closed questions are questions that can be answered with ‘yes’ or ‘no’ or with a very specific piece of information – have their place. But learners usually talk more willingly and freely in response to open questions because these convey the message that you are genuinely interested in the way learners see things.

Closed questions

Imagine putting questions like these to your supervisees?

- Do you know how to do the MUAC?
- Is it time for your visit?
- Will you be working in Kasarani next week?
- Would you consider working in Thika?
- Should I accompany you to your next meeting?

What are they likely to answer? Assuming that they are reasonably cooperative, they will most probably answer ‘yes’ or ‘no’ or give you a specific piece of information because these are all closed questions.

Closed questions are useful when you want:

- a ‘yes’ or ‘no’ answer;
- very specific information;

- to establish agreement;
- to check something before going any further

They are not helpful when you want to invite learners to talk about themselves and their experiences. If you start a conversation with a series of closed questions, you could be setting expectations that you will do all the thinking and talking.

Open questions

If you want a learner to talk more widely, use open questions.

- What happened?
- What's going on here right now?
- When you think about going to Mama Njeri's home, how do you feel?

Open questions can be answered in many different ways. They encourage people to:

- clarify their thinking;
    - "When you say '....', what do you mean?"
    - "You say he's got something against you. What would be an example of that?"

- look at the assumptions they might be making;
    - "What might be a reason for her doing that?"
- think about the implications of what they think, say and do;
    - "If you say that to your friend, how do you think she might react?"
- consider other viewpoints or perspectives;
    - "How is what you've just said different from what your colleague said?"

'Why?' is a tricky question to ask, it can sound confrontational and aggressive? Guard against this by using a gentle tone of voice.

Sometimes a person may not open up and answer with 'don't know' or 'nothing' You can use words such as 'tell me', 'explain' or describe to move the conversation in a particular direction while still giving the learners room to say what is on their mind.

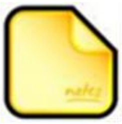

Facilitator's notes

Communication

It is exchanging information.

Communication is a two way process. It is the act of transmitting information, thoughts, opinions and feelings through speech, signs and actions from a source to a receiver. For a supervisor there is need to communicate effectively with the community health service provider otherwise the message, no matter how good, will not be heard or heeded. The communication process consists of a message, source, channel, receiver, effect and feedback. Communication begins with a message that is developed at the source. The source channels the message to the receiver. This message has an effect which produces feedback.

The flow of communication between a supervisor and a supervisee needs to be smooth and clear – not only in regard to the content, but in the way the content is conveyed. When people communicate, they take in a number of messages apart from the spoken words. A supervisor's communication role also includes passing information from top management to the community health service providers.
Listening is equally important, if not, more in communication as sending the message. Making sure that what we think we heard is indeed what the other person said is an essential part of effective communication. Also, it's important to talk how listening and communication applies to all types of relationships, between couples, co-workers, parent and child etc.

Session 2.2: Group Facilitation Skills

Group supervision involves a group of community health service providers coming together with a supervisor and conducting regular supervisory activities such as: data collection and interpretation, problem solving, and continuing education.

The advantages of this method is that it takes a team approach which has been shown to be more effective for many contexts, it relieves the 'solitude of working' and helps community health service providers to feel like part of a bigger process and working towards common goals. The notion of 'peer pressure' is relevant too, as public presentation of data and progress amongst team members, can lead to public recognition of their efforts and is a disincentive for low performance. From the groups, typically more competent members will emerge, who can be strengthened to provide additional support to the community health service providers – become 'lead providers' over time, taking on roles such as data audit / support, trouble shooting, and conducting observational assessment or case evaluations in the community (after their skills are well developed).

The peer-to-peer learning process which inevitably emerges during group approaches also has a huge value add to the project. Over time, community health service providers will become true experts in delivering services and many will encounter and overcome problems for themselves using local knowledge. This peer support may be a better source of solutions for overcoming cultural and behavioural barriers to health than that of the supervisor themselves.

The group approach, however, comes with limitations, key among which is the inability to identify falsification of data and observation of a home visit.

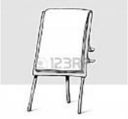

Activity one: Group dynamics- *Characters in a group (90 minutes)*

- Facilitator to give the participants a hand out on different animals that portray different characters in a group
- Participants are asked to share in a round which animal they identify with
- Facilitator to then share a hand out that has the characters explained and hold a discussion

with the participants
- Facilitator to conclude that this exercise is supposed to offer insight into understanding the group better. Every participant in the group will present with a different character and at times unhelpful behaviours that the supervisor will need to manage effectively.

How do you deal with unhelpful behaviour?

However, emphasis should be placed on the helpful behaviour in a group which are more frequent. People play different roles in different groups, one may be a natural leader but if they find someone else is better known or outgoing in the group they may start to play the joker or become the questioner (because there is a play of power (unconscious) or they may become supportive.

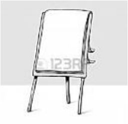

Activity two: Group dynamics- *Johari's Window model (30 minutes)*

- Facilitator introduces participants to Johari's window and explains its origin and quadrants in a flip chart.
- Facilitator invites participants to fill in the different quadrants aspects of themselves as described in Johari window model.
- Facilitator indicates that a fully functioning personality has a wide free area and that The aim in any group ***should always be to develop the 'Free area' for every person, because when we work in this area with others we are at our most effective and productive, and the group is at its most productive too. The open free area, or 'the arena', can be seen as the space where good communications and cooperation occur, free from distractions, mistrust, confusion, conflict and misunderstanding'*** invites participants to share in the group how through group interaction the free area can be widened
- Facilitator uses the diagram below to illustrate to participants how self – disclosure, feedback, insight etc. can be used within a group context to enhance personal growth and effectiveness in team work

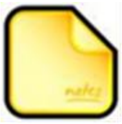

Facilitator's notes

The Johari Window model is a simple and useful tool for illustrating and improving self-awareness, and mutual understanding between individuals within a group. The Johari Window tool can also be used to assess and improve a group's relationship with other groups. The Johari Window model was developed by American psychologists Joseph Luft and Harry Ingham in the 1950's, while researching group dynamics.

The four Johari Window perspectives are called 'regions' or 'areas' or 'quadrants'. Each of these regions contains and represents the information - feelings, motivation, etc - known about the person, in terms of whether the information is known or unknown by the person, and whether the information is known or unknown by others in the group.

Johari Window four regions:

1. what is known by the person about him/herself and is also known by others - **open area, open self, free area, free self, or 'the arena'**
2. what is unknown by the person about him/herself but which others know - **blind area, blind self, or 'blindspot'**
3. what the person knows about him/herself that others do not know - **hidden area, hidden self, avoided area, avoided self or 'facade'**
4. what is unknown by the person about him/herself and is also unknown by others - **unknown area or unknown self**
5. When a participant enters a group or during the initial phases of group formation all members **free area** is small, through the processes of **self-disclosure** and **feedback** a participant is helped to widen the free area as is illustrated below.
6. **‘The aim in any group should always be to develop the 'Free area' for every person, because when we work in this area with others we are at our most effective and productive, and the group is at its most productive too. The open free area, or 'the arena', can be seen as the space where good communications and cooperation occur, free from distractions, mistrust, confusion, conflict and misunderstanding’.**
7. The common principle is that as the group matures and communications improve, so performance improves too, as less energy is spent on internal issues and clarifying understanding, and more effort is devoted to external aims and productive output.

Johari Window model

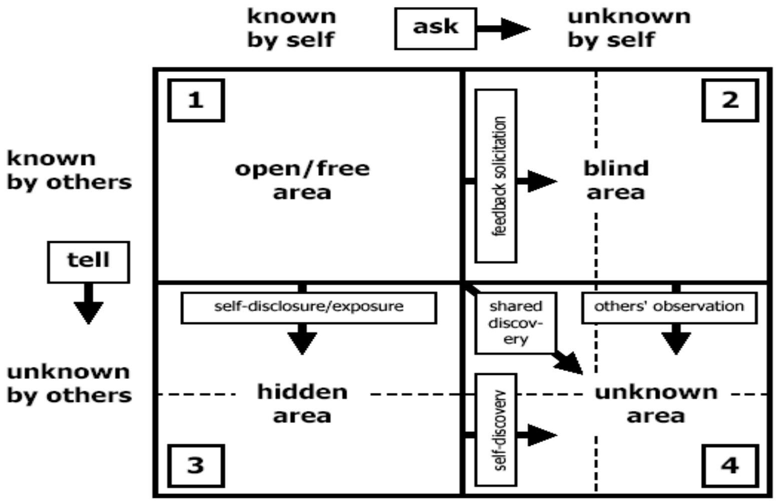

Emphasis needs to be placed on giving feedback in Johari’s window. Constructive feedback helps us to become more aware of what we do and how we do it. It gives us an opportunity to change and modify in order to become more effective communicators.

Effective feedback

- Be specific rather than general-Providing specific examples helps the community health service providers understand exactly what the issue is. You can then agree on the details and work on solutions:

Don’t say: “Your performance is below what I expect”
Say: “Your monthly report is not complete:
Don’t say: “Good”
Say: “The way you helped that pregnant woman with her referral was very good”

- Should include both positive and negative observations
- Where possible negative feedback should be sandwiched between positive comments; ascertain that the receiver hears positive as well as negative
- Feedback is focused on behaviour rather than on a person-what the person does rather than what we imagine the person is.
- Feedback takes into account the needs of the receiver of feedback.
- Feedback is about behaviour somebody can do something about
- Share information rather than giving advice.
- Feedback should be well timed and as immediate as is possible and appropriate. It should be given as soon after the event as possible.
- Give no more information than the receiver can handle.
- Do not guess or assume a motive.
- Check that the feedback is well understood.

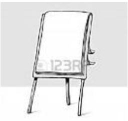

Activity three: Stages of group formation (30 minutes)

- Facilitator to write the following words in a flip chart
    - Forming, storming, norming, performing, mourning
- Facilitator to ask the participants to explain their understanding of each word in the context of a group
- The facilitator can then use a hand-out to explain each stage to the participants and what happens in each stage and how to deal with issues

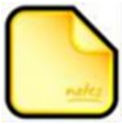

Facilitator’s notes

Forming stage (stage of uncertainty & confusion)

This is the initial entry of members to the groups, concern’s including:

- Getting to know each other
- Discovering what is considered acceptable behaviour
- Determine the group’s real task
- Why are we here? What are we doing? What is our purpose?

Storming stage (stage of conflict & confront)

A period of high emotionality and tension among group members, concerns include:

- Formation of coalitions and cliques

- Disagreements and arguments arise
- Clarifying membership expectations
- Dealing with obstacles to group goals
- Understanding members interpersonal styles

  Supervisors of the team during this phase may be more accessible, but tend to remain directive in their guidance of decision-making and professional behaviour. The team members will therefore resolve their differences and members will be able to participate with one another more comfortably. The ideal is that they will not feel that they are being judged, and will therefore share their opinions and views. Normally tension, struggle and sometimes arguments occur. This stage can also be upsetting.

Norming stage (Stage of 'we' feeling)
This is the point at which the group really begins to come together as a coordinated unit and having one goal, concerns include;

- Holding the group together – people know where they fit in and may have to give up their own ideas and agree with others to make the team function.
- Dealing with divergent views and criticisms
- Conflicts are resolved and there is a stronger sense of belonging to the group
- What kind of behaviours and contributions are acceptable and normalized within the group?
- Norms create structure- what do we stand for?
- Group can readily slip back into storming stage

Performing stage
It marks the emergence of a mature, organized and well-functioning group, concerns include;

- Group is in high gear and highly productive – work and progress commences on the basis of a relatively stable group structure
- Group members can now focus on the task and care for other members of the group
- Members deal with complex tasks and handle internal disagreements in creative ways and means that are acceptable to the group
- Primary challenge is to continue to improve relationships and performance
- Team cohesion and identity
- Results are observed in this stage

Mourning stage
A well-integrated group is:

- Recognising the sense of loss felt by group members

  Termination stage
- Separation and ending from tasks and members
- Able to disband when its work is finished
- Willing to work together in the future

The purpose of understanding the stages of group development helps supervisors realise that these stages are necessary for the group members to grow, face challenges, tackle problems, find

solutions, plan to work and deliver results. Once, the supervisors understand each stage they are able to develop a safe environment to learn, review achievements, and look at barriers that are hindering them from learning and looking at possibilities for improving.

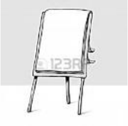

Activity four: Group facilitation skills (60 minutes)

- Facilitator to brainstorm with participants on some of the facilitation skills they may be aware of.
- Facilitator to write the responses on a flip chart and then fill in the gaps with information below and give examples

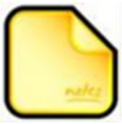

Group Facilitation skills

1. **Group focusing skill** - This means re-directing the client when they deflect from the topic of issue. It is asking the client for priorities of exactly what he/she would want to tackle at a particular time. It gives direction to the session.
2. **Facilitating –** opening up clear & direct communication within the group; helping members assume increasing responsibility for the group's direction.
3. **Initiating –** promoting participation and introducing new directions in the group
4. **Blocking –** intervening to stop counterproductive group behaviour
5. **Modelling** – demonstrating desired behaviour through action
6. **Terminating** – preparing the group to a close session or end its existence
7. **Giving feedback** – expressing concrete and honest reactions based on observation of members behaviours
8. **Supporting** – providing encouragement and reinforcement
9. **Clarifying** – grasping the essence of a message at both the feeling and thinking levels; simplifying clients statements by focusing on the core of the message
10. **Evaluating** – appraising the on-going group process and the individual and group dynamics.
11. **Setting goals** - planning specific goals for the group process & helping participants define concrete and meaningful goals
12. **Active listening** – it is attending to verbal and non-verbal aspects of communication without judging or evaluating
13. **Restating** – paraphrasing what a participant has said to clarify its meaning making clear to the community health service providers that you understand them like in group dynamics
14. **Empathizing** – identifying with clients by assuming their frames of reference e.g. work environment, challenges
15. **Confronting** – challenging members to look at discrepancies between their words and actions or their bodily and non-verbal messages; pointing to conflicting information or messages
16. **Summarizing** – pulling together the important elements of an interaction or session

17. **Questioning** – asking open ended questions that lead to self-exploration of the "what" and "how" of behaviour.

Session 2.3: Problem solving skills

Problem solving is one of the skills required of a supervisor. Unfortunately most supervisors lack skills to effectively solve problems. Problem solving skills entails analysis and identification of problems for them to be understood and solutions to be formulated and implemented.

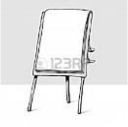

Activity one: Problem identification and analysis (30 minutes)

- Facilitator to lead the participants in a discussion to define what a problem is and develop a working definition for it.
- Facilitator to ask the participants to provide a problem list facing their work in community health strategy program. Facilitator writes down the identified problems
  These should be guided by what was evidenced by the findings of the context analysis of the program e.g. lack of supply of tools for data collection, inadequate fuel for transport, lack of bicycles for CHWs and motorbikes for CHEWs, over expectations from the community, insecurity for providers, high workload for CHEWs, lack of incentives for volunteering CHWs, etc.
- Facilitator then brainstorms how they identify problems which affect their performance and that of their supervisees. Write responses on a flip chart.
  This can be through concerns raised by supervisees, through reports e.g. routine reports or reports from studies done, interpreting results of an activity, through brainstorming meetings. Some problems can also be pointed out by the community and other stakeholders who are not directly associated with the health system
- Facilitator to explain to participants the importance of prioritizing problems. Begin by saying "as a supervisor it is important to refrain from tackling all the problems at the same time". Facilitator to ask participants what they would consider during prioritization of problems and lists them on a flip chart.
  This can be done by taking the following into consideration:
  - *Seriousness: the problem in relation to organization's/ supervisee well-being i.e. which problem will have the greatest impact if tackled first?*
  - *Availability of resources: e.g. staffing levels, equipment, time, drugs, money, etc.*
  - *Feasibility/ practicability: i.e. how much control does the supervisor or the team have over addressing the problem? Where the solution is outside the team's mandate support should be sought from the next level of authority.*

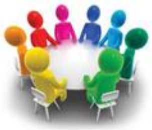

Activity two: Problem prioritization (25 minutes)

- Facilitator to divide the class into groups of 5-10 and provide each group with a flip chart paper. The groups should have leaders who will ensure that action plans developed at the end of this module section are carried through by the persons responsible for actions plans which will be developed.
- Introduce the problem matrix below and explain that it can be used if one is experiencing difficulties in prioritizing problems

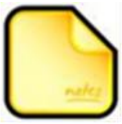

Facilitator's notes

Problems	Seriousness in relation to community health<br>3= Very Serious<br>2= Quite serious<br>1= Not serious	Cost of resolving the problem<br><br>3= Low<br>2= Medium<br>1= High	Practicability<br><br>3= Very easy<br>2= Quite easy<br>1= Not easy	Total Score
e.g. Lack of motorbikes				
Lack of incentives for CHWs				
Over expectations from Community				

- In their groups ask participants to draw the problem matrix and after discussing list the first five priority problems in the matrix from the list developed in Activity one above. Each individual in a group then takes part in the problem prioritization by entering a score in each of the column as illustrated below.
  NB: Scoring is systematically done per column and one has to explain his/ her scores to the group members, i.e. begin by scoring for all items under seriousness before moving to cost.

Problems	Seriousness in relation to community health<br>3= Very Serious<br>2= Quite serious<br>1= Not serious	Cost of resolving the problem<br><br>3= Low<br>2= Medium<br>1= High	Practicability<br><br>3= Very easy<br>2= Quite easy<br>1= Not easy	Total Score
e.g. Lack of motorbikes	3+3+3+2+2	1+1+1+1+1	2+2+1+1+3	27
Lack of incentives for CHWs	3+3+3+3+3	1+1+1+1+1	3+3+3+2+2	30
Over expectations from community	2+2+2+2+2	3+1+3+1+1	3+3+3+2+2	32

- Facilitator asks each group to present their matrices to the plenary.

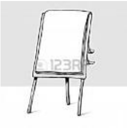

Activity three: Problem Analysis (25 minutes)

- Facilitator leads the participants in listing problems scored from all the groups from those with high scores to those that scored low points.
- Facilitator asks the participants to go back to their groups to each pick a problem starting from the top e.g. if there are two groups, each group should pick from the first two problems.
- Facilitator guides the group in conducting a problem analysis by selecting from the list a problem that has not been picked by either of the groups

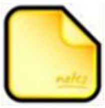

Facilitator's notes

Problem analysis is dissecting a problem in order to understand how it emerged. It helps in understanding the root cause of a problem. When analysing problems one should:

- Use a problem tree
- Begin by identifying the principal causes
- Be honest
- Brainstorm on why the problem is happening
- Concentrate on the common causes
- Keep asking 'but why?' until you have identified the root cause

The question but why should be asked for each of the causes until it can no longer be answered/ ask.

Example of a root cause analysis (ref: REACHOUT Kenya Phase 1 report):

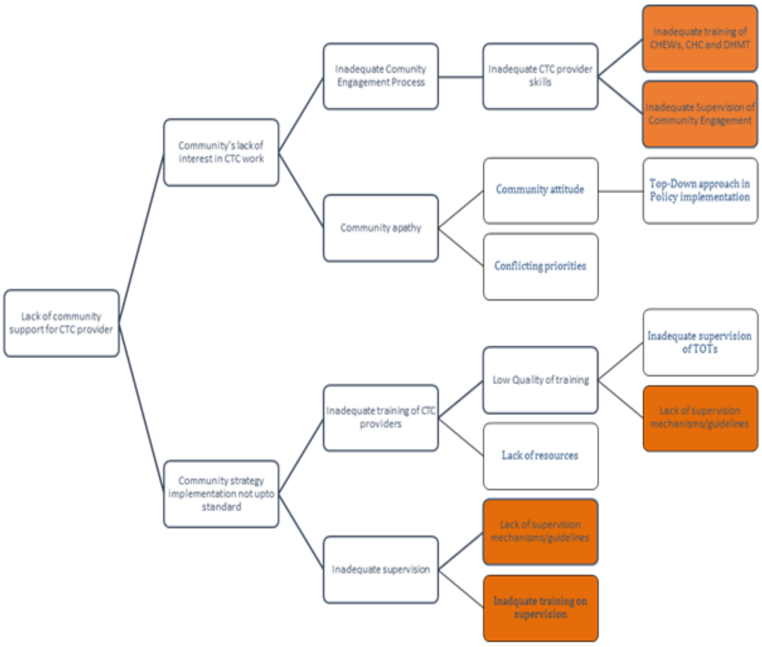

Problem analysis assists in defining a problem and identifying target areas for intervening in order to resolve the problem

- Each group is asked to conduct a problem analysis of their selected problems and present back to the plenary

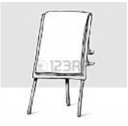

Activity four: Problem Definition (25 minutes)

- Facilitator leads the group to develop statements for their problems. Facilitator to explain that the statements need to be simple (without jargon); precise (to the point); quantified (using numbers or percentages); and negative (for the extent of the problem to be felt)

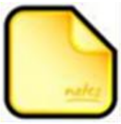

Facilitator's notes

A persuasive problem statement consists of three parts: A) the ideal, B) the reality, and C) the consequences (ref. professorbwisa.com).

A. the ideal: Describes a desired goal or ideal situation; explains how things should be.

B. the reality: Describes a condition that prevents the goal, state, or value in Part A from being achieved or realized at this time; explains how the current situation falls short of the goal or ideal.

C. the consequences: Identifies the way you propose to improve the current situation and move it closer to the goal or ideal.

There are four steps to writing a problem statement. Guide participants using one of the problems identified e.g. over expectations from community

Step 1

- Begin by describing a goal or desired state of a given situation, phenomenon etc. This will

build the ideal situation (what should be, what is expected, desired)

- Example: Community Health Workers (CHWs) need support from the community in order to perform their duties.

Step 2

- Describe a condition that prevents the goal, state, or value discussed in step 1 from being achieved or realized at the present time. This will build the reality, the situation as it is and establish a gap between what ought to be and what is.
- Example: There is however too much expectations for CHWs from community members. The community member asks for things such as treatment and financial support from the CHWs which is not within their mandate to offer.

Step 3

- Using specific details show how the situation in step 3 contains little promise of improvement unless something is done.
- Example: Since the CHWs have not been able to meet these kind of expectations from community members the later have continually felt that their needs are not fully met. Despite efforts by the CHWs to explain the scope of their services some community members have rejected services feeling that the providers are intentionally refusing to give them what they need. If this persists the number of households rejecting services from CHWs will grow in number which will affect the main goal of establishment of community health services i.e. ensuring universal access.

Step 4

- Then emphasize the benefits of how you plan to intervene by projecting the consequences of possible solutions as well.
- Example: There is need to explore various community engagement options through which dialogue can be done by community members to explain the importance and scope of CHW work in order to manage their expectations. This will encourage community support and acceptance of CHW work

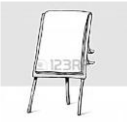

Activity five: Suggesting Solutions for Problems (25 minutes)

- Facilitator to ask the group to suggest solutions on how to solve the problems identified
- Facilitator to write the responses on a flip chart and then fill in the gaps with information below

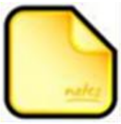

Facilitator's notes

It is important to explain that it may not always be possible to find a solution to every listed root cause. Developing solutions should be a team effort. Choice of solutions depends on:

- Availability of resources
- Feasibility/ Practicability
- Whether it addresses the root cause. Try tracking the cause which the solution is tracking from the problem tree, ask yourself whether intervening on it will result in resolution of all

the other causes attached to it

- Community support
- Management support

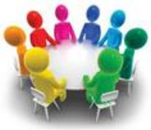

Activity six: Developing target statements and a change plan (25 minutes)

- Facilitator to ask the group to develop target statements for their solutions and present to the plenary.
- Fill in the gaps with information below and give examples

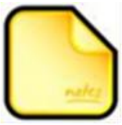

Facilitator's notes

A target statement is a sentence depicting what is intended to be achieved in order to improve on an identified problem. Target statement should be:

- SMART (Simple, Measurable, Achievable, Realistic, Time bound)
- Positive

It describes the extent to which the team aims at solving the problem e.g. All CHEWs in Community Units at sub-county XXX will develop and execute community engagement plans with CHCs targeting sensitization of community members on roles and responsibilities of CHWs by Dec 2014.
Groups should avoid ambitious statements as they will experience disappointment if the targets are not met.

- Facilitator then asks the groups to identify activities needed to arrive at solutions identified in Activity 5 above. The groups should populate their ideas in a table like the one below:

Problem Identified: 48% of the clients visiting Better Living VCT Centre between January and March 2010 do not receive a condom demonstration				
**Target:** 80% of the clients visiting Better Living VCT Centre between June and August 2010 will receive a condom demonstration				
**Activities (towards the solution)**	**Resources needed**	**Timing (dates)**	**Evidence activity is completed (monitoring mechanism)**	**Persons Responsible**
Community meetings / Mobilization to clarify myths & misconceptions about condoms	• Staffs time<br>• Transport	20th April & other dates	Reports of the meetings attended / conducted	Counselor<br>Other staff<br>Community health volunteers
Condom demonstration posters in the VCT room and office cubicles	• Staff time<br>• Condom demonstration<br>• Posters	23rd April	Poster in display in the counselling room and in the office (cubicles)	Counselor
Penis model to be displayed in the VCT room as a reminder to the counselor	• Penis model with a stand<br>• Staff time	23rd April	Penis model in the VCT room	Counselor
Deal with counselor values through supervision and self assessment	• Counselors time<br>• Supervisor<br>• Self assessment forms	25th April	Supervision (counselor and record of counselors attendance for supervision	Counselor and supervisor

It is important to assign people who will be responsible for each assigned activity and spell out exactly how they will monitor (evidence) that the activity has been completed. When preparing plans remember:

- To clearly specify roles and responsibilities for each activity
- Avoid assigning all activities to the same person(s)
- Decide on who is/are the most appropriate person(s)
- Plan within the limits of available resources
- Not to attempt to tackle problems which are outside the teams' control

Facilitator to ask the groups to present their plans to the plenary
The group needs to understand that the execution of this plan in the field will be part of their training evaluation by the facilitators.

Session 2.4: Advocacy skills

Advocacy for community health services

Advocacy is an example of a change process which targets people who have influence over others e.g. decision makers, leaders, and policy makers. It aims at changing policies, laws and practices. It can be used to solve problems faced by community health service providers. Advocacy is about influencing people and organizations in power to create an environment which supports their work. Supervisors require capacity and skills in advocacy in order to influence decisions on issues affecting their work directly and that of supervisees.

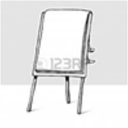

Activity One: CHEWs experience in advocacy work (20 minutes)

- Facilitator to ask participants whether they have been done advocacy for community health work and how they did/ have been doing it.
  Have a discussion with the group on their experiences – what was the problem, who was involved in the advocacy, what was the objective, who was the advocacy directed at, what methods were used, what difficulties were faced an how they were overcome, what were the results of the advocacy, what sources of support did they find most helpful, what were the lessons learned?

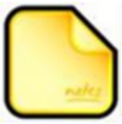

Facilitator's notes

The Advocacy Framework

The advocacy framework used here is borrowed from the one developed by the International HIV/AIDS Alliance which was an adaptation from a framework that was earlier developed by International Council of AIDS Service Organizations (ICASO)

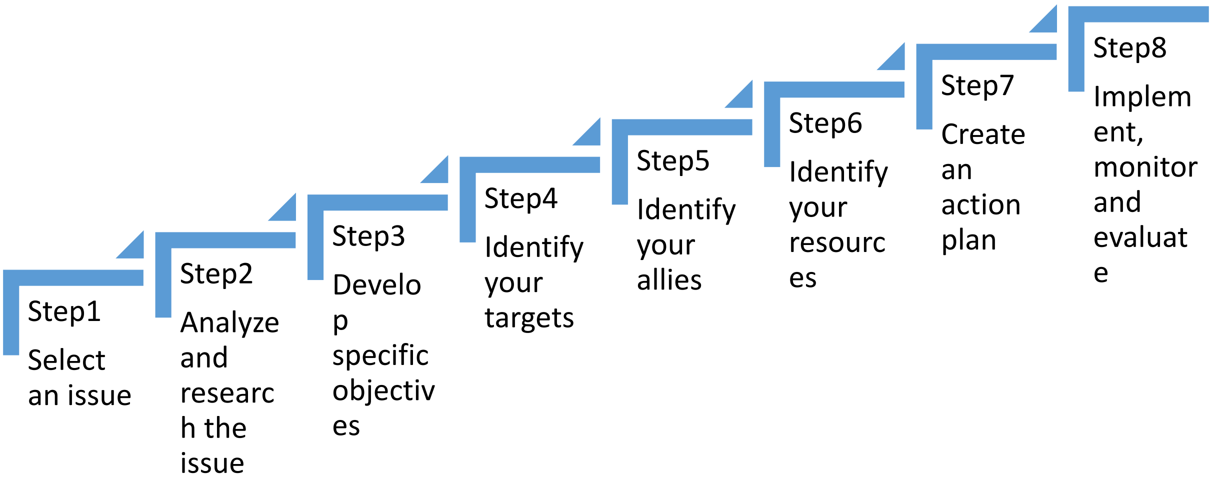

Explain to participants that they will practice using the framework above in their groups by applying it to their chosen issue. The participants also need to appreciate that the Steps will be realistic in order to produce a living document by Step 7.

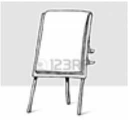

Activity two: Identification of issues that require advocacy (20 minutes)

- Facilitator to maintain the groups selected at Problem Solving and provide each group with a flip chart paper
- Ask each group to each identify one problem that require advocacy from the list of problems that had earlier been developed.

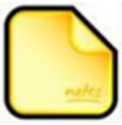

Facilitator's notes

Participants should discuss in their groups whether the issues selected can only be resolved through advocacy or whether advocacy will complement the problem solving process. Participants need to first ask themselves "Should this issue be resolved by advocacy" before moving to "Will there be benefits to the community".

NB: Facilitator should emphasize that using evidence from literature such as reports and scientific papers increases the chances of their solutions/ ideas being taken into account by decision makers. Mention that they can ask for reports and papers from organizations dealing with community health work e.g. the LVCT Health report on the Context Analysis of CHW work has evidence of challenges faced by CHWs. For class practice the participants may not have evidence but they should have their problem statements with references by the following day of the exercise.

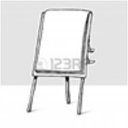

Activity three: Step 4 and 5- Identification of targets and allies (20 minutes)

- Facilitator to introduce the participants to the Venn diagram below to assist them identification of advocacy targets:

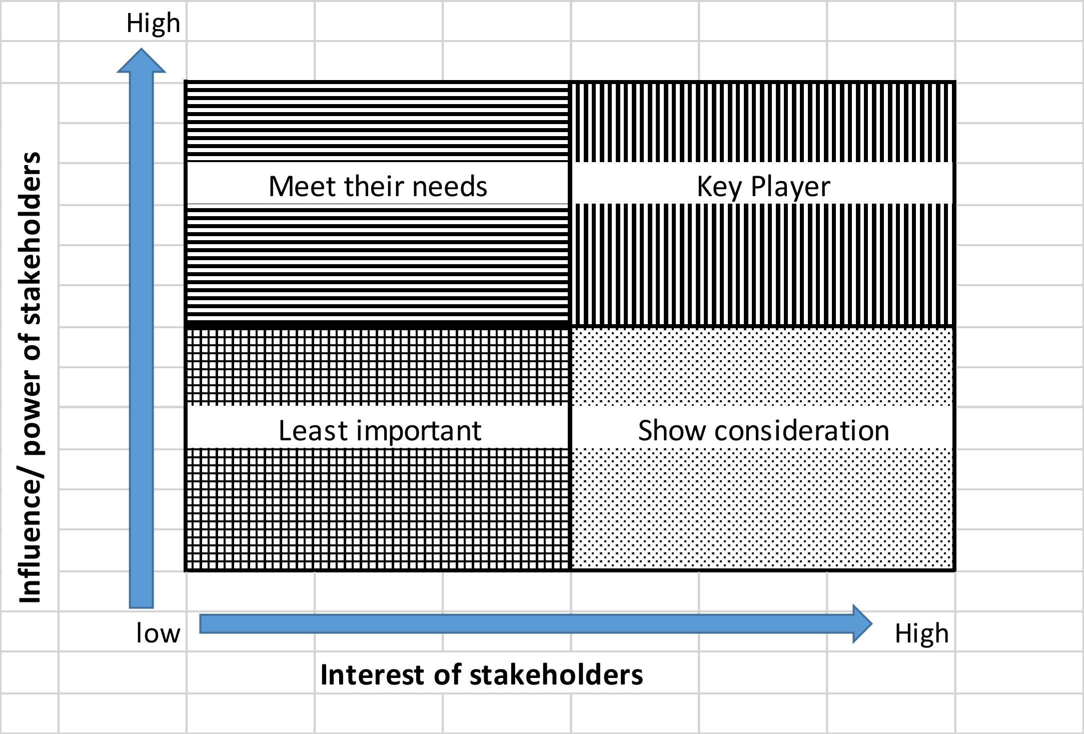

- Facilitator to ask each group to list all the stakeholders who can make their advocacy efforts a reality in each section above.

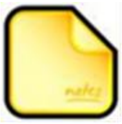

Facilitator's notes

These decision makers include individuals from both government and non-governmental organizations. Each section should have two lists of those they can advocate to and those that they can advocate with. The lists should be very specific with names attached to them e.g. The Head of the Community Health Unit – Dr. XXXX, the County XXXX CHS Coordinator – Mrs. XXXX, the County Health Director of NGO XXX – Dr. YYY, the CHEWs of Sub-county MMMM – Miss WWW, Mr. NNNN, etc. The list of designation and names should be created under each title in the four sections of the diagram above.

Groups should keep in mind what is happening outside their organization that may impact their

strategy e.g. potential opportunities and barriers, activities of other organizations.
Conducting an external scan will influence the type activities and plans to be made in achieving objectives including the venue and timing of activities. It may also provide guidance on who are the allies and the targets. One may take advantage of the available platforms and timing to carry out their activities or will put safety nets to safeguard on foreseeable barriers.

Identification of targets: A target is an individual or an organization who is advocated to. Groups to ask themselves who makes their objective a reality by taking a specific action or changing a specific behaviour. There is need to think critically about who to target e.g. target directly those with influence vs. target those who can influence the people with influence. The target will determine the advocacy method and/ or activity. From the Venn diagram the best targets are the Key Players however those with high power and low interest (in the 'meet their needs' section) can also be targeted in advocacy.

Identification of allies: Allies are those we advocate with. There are natural allies who are interested in the same issue and understand the roles played by all in tackling the issue. Allies can also be the indirect targets who may be sympathetic to your objective or may have influential people but have undergone some influencing for them to support the change. One can form a coalition with allies and this can result in achieving more compared to working individually. However this requires resources and might be difficult yet produces the most rewarding results. Coalitions can be short term or long term. From the Venn diagram the best allies are again the Key Players however those with low power and high interest (in the 'show consideration' section) can also be approached to be allies.

Step 7 _ creating an action plan

- Facilitator explains the objective of the step which are two i.e. to select appropriate advocacy activity and to develop detailed plans for activities chosen.

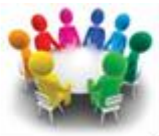

Activity 4: Selecting appropriate advocacy activity (20 minutes)

- Facilitator to introduce various advocacy tactics and how they are generally employed *(see the next table below in the tactics column for ideas)*
- After explaining the various tactics, facilitator to introduce the table below. Give a copy to each individual and ask them to complete for each tactic under the various column as high or low.
- In the plenary ask the team about their general thoughts of the table and what individuals scored under the columns.

Explain to the team that the best tactic is that which has a high impact on both the decision makers organization, low risk on the organization, and does not require high resources.

Think Inside the Box....

Tactics		Impact on decision makers	Impacts on the organization	Risks on the Organization	Resources it requires
1	Petitions/Marches/Strikes				
2	Videography				
3	Media mobilization				
4	Direct lobbying (meetings with authorities)				
5	Indirect lobbying (mediators)				
6	Reports, research material, fact sheets and briefs				
7	Charter/Declaration				
8	Workshops				
9	Forum/Debates (national & international)				
10	Door to door approach				
11	Opinion leaders as spokespersons				
12	Online Communication (social media/ website news/blogs)				
13	Newsletters and Magazines				
14	Academic Journals				
15	Performing Arts (Community Theatre, Song, Poetry, Drama)				
16	Video and Audio				
17	Posters and publicity material				

Facilitator's notes

Selecting appropriate advocacy methods:

Advocacy objective:	To persuade managers of the 10 largest companies in the Andhra Pradesh state to end compulsory testing of workers and dismissal of HIV+ workers.
Direct targets:	General managers of companies.
Indirect targets:	Labour unions, boards of directors, personnel managers.

Method	Strengths	Weaknesses
Analysing and influencing legislation and policies or their implementation	If analysis shows that a company's current practices are costing them money, this can be powerful evidence<br>Beneficiaries can provide expertise	Criticism of policies could anger managers<br>Not useful for managers who dislike formal policies
Position paper or briefing note	Suitable for presenting to senior directors and managers<br>Useful background briefing for journalists<br>Ensures that public statements by allies always agree	Can easily be lost among other paperwork<br>Some managers do not like reading papers<br>Difficult to involve beneficiaries
Working from inside	Some managers will listen more closely to people they know<br>Many opportunities within labour unions	Limited opportunities in companies – all policy is made by managers and directors
Lobbying or face-to-face meetings	Opportunity to present 'human face' of the issue and to build a personal relationship<br>Beneficiaries can explain their case directly	Managers often too busy to attend<br>Board members not interested in the issue, and afraid of HIV+ people
Presentation	Opportunity to present the issue in a controlled way, direct to decision-makers<br>Beneficiaries can speak directly	Managers often too busy<br>Difficult to gain permission for presentation to board of directors
Drama	Emotional appeal works with some managers<br>Suitable for mass meetings of labour unions<br>Beneficiaries can advise on story, or perform	Some decision-makers will feel that drama is only for the illiterate<br>Difficult to find opportunity to perform to managers or directors
Press release	Useful for organisations needing public support<br>Useful to launch a campaign or for quick reaction to opposition or new developments<br>Inexpensive	No use for companies who do not need/want public support<br>Difficult to involve beneficiaries
Media interview	Same as for press release<br>Useful at times when advocacy issue needs 'a human face'<br>Inexpensive	Can have negative impact if the interviewee is not prepared or does not deliver message well<br>Can be manipulated by journalists
Press conference	Same as for press release<br>Good for presenting evidence, esp. case studies/examples<br>Useful to launch a major campaign or for reaction to serious opposition or major new developments<br>Easy to involve beneficiaries and allies, and give them public recognition	As for press release<br>Requires high level of organisation<br>Expensive

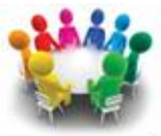

Activity 5: Mapping a plan (20 minutes)

- Facilitator to introduce a Target Information Table (see below) and ask groups to list and conduct an analysis of the targets identified at Step 4 above.

Target	How to Contact	Target's feelings about the advocacy issues -ve/ +ve	How to influence the target	Target's way of making decision	Target listens to (possible indirect targets)

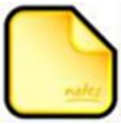

Facilitator's notes

Facilitator should explain that advocacy activities should be determined based on the target audience. Have groups populate their table like the one below.

Target information table

Target	How to contact the target	Target's feelings about the advocacy issue	How to influence the target	Target's way of making decisions	Target listens to (possible indirect targets)...
The President of the Republic of Zimbabwe	With a letter<br><br>Through the President's office	Negative	Majority rule	Through Parliament<br><br>Unilaterally	Ministers<br><br>Governors
Dr T. Stamp	Through the Ministry of Health<br><br>Through letters, meetings and ceremonies	Supportive	NGOs<br><br>Argument of 'good health delivery system'	Consultation	NGOs<br><br>Ministry of Health personnel
Governors<br><br>Provincial AIDS Councils (PAC)	Through direct approach<br><br>Through visits, phone calls, meetings, invitations to functions	Negative	NGO achievements<br><br>Stakeholders' feelings	Through PAC or District AIDS Councils (DAC) Committees	Local traditional leaders<br><br>NGOs
P. Misihairambwi	By phone, e-mail, through interviews	Very supportive	Experience as an NGO activist<br><br>Her post in Parliament	Through contacts with influential people	NGOs<br><br>Donors<br><br>Individual activists

Reference: Adapted from an advocacy skills-building workshop for HIV/AIDS, International HIV/AIDS Alliance, Zimbabwe, July 2001.

Depending on each target's feelings about the advocacy issue, groups need to ask themselves whether they need a plan that will frame, fortify and amplify, or reframe.

Frame aka sharing knowledge – target is not talking about the issue or has no idea about your issue
Fortify and amplify aka building will – A positive action is being taken over the issue, you like the action and want to push it further
Reframe aka reinforcing action - An action is being taken over the issue, but you want to change the direction that it is taking

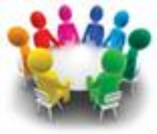

Activity 6: Developing a detailed plan for activities (20 minutes)

- Facilitator to ask groups to design an action plan using the Table below. The groups should consult their work from Steps 1 – 6.
- Facilitator to ask groups to add indicators (definitions of success/ desired change) that will demonstrate whether their advocacy objectives are being met.
- The action plans should be shared with the facilitators and copies made for follow up by facilitators at field

Objectives	Targets	Activities	Resources Required	Persons or organizations responsible	Timeframe	Outputs	Expected Outcomes	Indicators

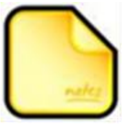

Facilitator's notes

An action plan must be realistic, have a specific set of activities with time lines and show who is responsible for implementing each activity. It should include both informal and formal activities e.g. having lunch with targets. It should also show what objectives each activity is specifically answering to. Unlike the previous steps above groups should coordinate their advocacy activities since they are all working towards the efficiency of the system e.g. if they have the same targets or similar activities. This will avoid duplication of efforts, enhance internal coalition and save on resources.
As a group is presenting back to the plenary, aside from giving feedback, the other groups should seek to identify areas of possible coalition and update their action plans accordingly
Outputs – What will be produced to reach your objectives e.g. letters, e-mails, phone calls, meetings

Outcomes – What are the results of your outputs e.g. increased funding, hiring of new staff

members

Example of action planning:

Coalition: Family AIDS Caring Trust, Daranayi NGO, individual PLHAs, and Murambinda hospital
Advocacy aim: Encourage the community leaders in Gombahari to publicly support people with HIV/AIDS

Objectives	Targets	Activities	Resources required	Persons or organisations responsible	Timeframe	Expected outcome
Objective 1 By July 2002, three influential community leaders will make positive public statements defending people living with HIV/AIDS	All community leaders	Meeting with all community leaders of Gombahari on stigmatisation of PLHAs	Manpower: Team leader, community leaders<br>Stationery: books, pens etc. for record-keeping<br>Vehicle<br>Food<br>Money	Team leader of organisation	September 2001	Come up with three most influential leaders
	Three most influential leaders	One-to-one meetings with the three leaders who were most influential during the meeting	Team leader, community leaders<br>Money<br>Vehicle	Team leader	By early October 2001	Positive support from three community leaders willing to make public statements
	Community members	Meetings between three community leaders and their communities	Manpower: Team leader, three community leaders, PLHAs, community<br>Vehicle<br>Food	Team leader, community leaders	April 2002	Public statements defending PLHAs

Reference: Adapted from an advocacy skills-building workshop for HIV/AIDS, International HIV/AIDS Alliance, Zimbabwe, July 2001.

Example of a plan with indicators:

Example of Activity 2.8: By NGOs/CBOs based in Zimbabwe

Coalition: Family AIDS Caring Trust, Dananayi NGO, individual PLHAs, and Murambinda hospital

Advocacy aim: Encourage the community leaders in Gombahari to publicly support people with HIV/AIDS

Objectives	Targets	Activities	Resources required	Persons or organisations responsible	Timeframe	Expected outcome	Indicators
Objective 1 By July 2002, three influential community leaders will make positive public statements defending people living with HIV/AIDS	All community leaders	Meeting with all community leaders of Gombahari on stigmatisation of PLHAs	Manpower: Team leader, community leaders<br><br>Stationery: books, pens, etc. for record-keeping<br><br>Vehicle, Food, Money	Team leader of organisation	September 2001	Come up with three most influential leaders	Leaders:<br>– who are listened to by other leaders<br>– from whom local people ask for advice most often<br>– who have successfully led a change in policy or practice in the past<br>*Monitoring methods: informal survey, observation at meeting*
	Three most influential leaders	One-to-one meetings with the three leaders who were most influential during the meeting	Team leader, community leaders<br><br>Money<br>Vehicle	Team leader	By early October 2001	Positive support from three community leaders willing to make public statements	Leaders:<br>– show understanding of why stigma is a problem<br>– show understanding of how public support for PLHAs can help<br>– promise to make public statements in support of PLHAs<br>*Monitoring methods: NGO reps observe and discuss after meeting*
	Community members	Meetings between three community leaders and their communities	Manpower: Team leader, three community leaders, PLHAs, community<br><br>Vehicle, Food	Team leader Community leaders	April 2002	Public statements defending PLHAs	Number of positive statements<br>Number of people hearing statements<br>*Monitoring methods: NGO reps attend and observe meetings; informal surveys in community*
Objective 2							

Reference: Adapted from an advocacy skills-building workshop for HIV/AIDS, International HIV/AIDS Alliance, Zimbabwe, July 2001.

Module 3: Process of supervision

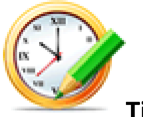

Time: 4hrs and 30mins

By end of this module, the participants will be able to:

1. Describe the components of the supervision framework for community health extension workers
2. Demonstrate ability to implement activities in each of the supervision components
3. Demonstrate ability to effectively carry out group supervision

Session 3.1: Introduction to the components of the supervision framework

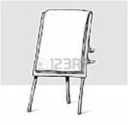

Activity One: Supervision functions (90 minutes)

1. The facilitator circulates a hand-out of the supervision framework to participants and takes them through the framework describing each components (see below)
2. Facilitator writes the title of each function below and a series of questions for each function on a separate flip chart.

   Flip chart 1: Educative function
   - How do the supervisors identify the capacity gaps
   - At what point do you identify the knowledge and skills gaps?
   - What do you do after you identify these gaps?
   - What concerns may emerge under the educative function of the supervisors

   Flip chart 2: Mentorship
   - What is mentorship (consider what it means to be a good or bad mentor)
   - Review two case studies (see hand-out) and determine which supervisor is providing mentorship and which one is not and give reasons
   - Why is mentorship important?

   Flip chart 3: Supportive function
   - How should you prepare for group supervision meetings
   - Outline the steps for carrying out Group Supervision
   - What concerns may emerge under the supportive function for community health service providers?
   - How do they debrief with each other?

3. The participants are divided into 3 groups. Each one is given one of the flip charts and is

asked to spend 40 minutes discussing it and writing out detailed answers on flip charts. Give the “mentorship’ group the case study hand out that accompanies this curriculum.

4. The groups report back their findings for discussion
5. Fill in the gaps using the notes below.

Supervision framework for community health services

IMPLEMENTATION

INPUTS

- Trained supervisors
- Community Health workers
- Community health service delivery commodities and supplies
- Support supervision tools
  - Group supervision meeting tool
  - Monthly assessment: one-to-one tool
  - Field observation supervision tool
  - Spot checks supervision tool
- Community health Data recording and reporting Tools (collation and reporting –to the top and down)

PROCESS (for Supervision and Quality Management)

Educative

- Capacity building based on identified gaps
- Mentorship and coaching for quality improvement

Supportive

- Monthly Group supervision/Debriefings
- One to one support supervision
- Client work presentation and discussions during group supervision meetings

Administrative

- Site support supervision/ spot checks
- Data record reviews and analysis and reporting to address performance gaps
- Routine Data Quality Assessments

OUTCOMES

- Sustained supply of community health services logistics – data recording and reporting tools
- Improved Community Health Services data processes including recording, reporting and utilization for quality improvement.
- Sustained and institutionalized implementation of support supervision for community health workers
- Sustained adherence to CHS related protocols and standard operating procedures
- Increased team spirit and team work in the implementation of the community [illegible]

FEEDBACK

Hand-out for flip chart activity: CHW looking for supervisor for help

Case A: During one household visit, the supervisor notices that a CHW is not using the MUAC properly. The CHW is giving the MUAC to a 4-month old baby. When the visit is finished, the supervisor sits down with the CHW. Instead of telling the CHW that she made a mistake, she describes what she saw, and then asks: "Why did you do it this particular way?" The CHW explained that because the child was under one year old, she has to take the MUAC. Hearing this, the supervisor was able to use the situation as a teaching moment. She explained that since the baby is under 6-months, and is exclusively breastfeeding, the baby is not eating food and therefore the MUAC is not needed.

Case B. A CHW reports to her supervisor after a particularly challenging household visit. During the visit, the mother was distracted by children who needed her attention, and when the woman got up to help the children, the husband – who had joined the conversation – told the CHW he wanted her to come back and sleep with him. The CHW told him, firmly, "that is not why I'm here." Although the husband acted as if it was no big deal, the CHW left the household visit upset. The CHW called the supervisor and talked about what happened. The supervisor said, "You should not worry about it. You have an important job to do. Do not be distracted."

Facilitator's notes <u>Supervision functions/components</u>

1. **<u>Educative function</u>**

Supervisors should be responsible for formal teaching, such as skills training, knowledge and information (theory base) and personal development of community health service providers by allowing them to reflect on their work with clients. The supervisors then fill in gaps in knowledge and skills and make practical what was only abstract knowledge but what must now become working knowledge. A link is established between theory and practice. This function has been called the 'formative' function of supervision.

It is done through:

- **Capacity building within the groups**

Supervisors can use the group supervision meetings to increase the knowledge of community health service providers in a particular area of service where gaps have been identified or by facilitating topics that are related to their work in the community.

- **Mentorship and coaching**

Mentorship is a system of practical training and consultation that fosters on-going professional development with an aim of yielding sustainable high quality community health services (National Mentorship Guidelines for HIV Services in Kenya 2009) Mentorship aims at promoting continuous professional growth and development amongst community health workers consequently resulting in improved quality of community services. Mentorship will be undertaken by supervisors (mentors),

with an objective of helping less experienced community health service providers (mentees) apply theoretical knowledge acquired from different learning fora to practice, build their capacity and motivate them to continuously provide quality services.
What concerns may emerge under the educative function for community health service providers?

Mentorship is not as easy as it sounds. It is not a one-off instructive activity, it takes time and patience.

- Mentorship is a supportive relationship between a supervisor and supervisee
- Mentorship requires a willingness to share skills, knowledge, and expertise
- Mentorship is a professional alliance where a mentor who is more experienced
- can provide a mentee guidance, advice, and assistance
- A good mentor takes a personal interest in the mentoring relationship and provides guidance and constructive feedback. S/he should get to know his/her mentee, what his/her mentee's goals are, and what his/her mentees strengths and weaknesses are
- A good mentor values the opinions and initiatives of his/her mentees
- A good mentor motivates others by setting a good example
- A good mentor is authentic –"walk the walk" as they say – meaning the mentor does what he/she teaches.
- A good mentor will genuinely listen to concern; know his/her mentee's projects by name and be able to ask him/her how things are going
- A good mentor can recognize his/her mentee's talent and potential, and work to uplift that mentee

Importance of mentorship

- Mentorship provides one-on-one coaching and support
- Mentorship provides coaching and counselling to a junior member of the team in handling frustration and disappointment, working through difficult situations, providing constructive criticism, and behaving with humility and compassion
- Mentorship offers employees professional and personal development, which in turn can lead to higher motivation and job satisfaction
- A mentor will hold a mentee accountable for his/her actions and can assist the mentee in setting realistic short-term and long term goals
- A mentor is an advocate for a mentee in the broader system
- A mentor is a trusted colleague to discuss problems and find solutions

Case A demonstrates a strong mentorship relationship. Rather than simply saying, "you did not do it well" the supervisor was able to learn what was motivating the CHW to use the MUAC, and by doing so, was able to correct a misunderstanding the CHW had developed about MUAC.
Case B response tells the CHW to do something differently, without explaining what or how

2. <u>Supportive Function</u>

<u>This refers to the types of supportive activities we talked about on Day 1: Group supervision, peer</u>

supervision, one-to-one supervision, spot checks and home visit support supervision. Focus on group supervision.

The word supervision is often used in health care to talk about the process of giving advice and support to health workers. However, the word tends to evoke thoughts of someone who is bigger, better, smarter – the 'in-charge'. A more effective type of supervision is **supportive supervision**, where the supervisor's job is to be of help and give suggestions, rather than disciplining health workers and making sure orders are followed. It may require that you, as a supervisor, start to think differently about what it means to be 'in-charge'. Though supportive supervision will take some practice, in the end, we believe it will produce better result. The effective supervisor pays attention to all conditions that enable a community health worker to grow and work to the best of her ability.

Community health service providers need support from outside the village, such as[1] in the supply of medicines, continued training, and a friendly relationship with a nearby clinic and hospital for when their patients need a referral. This support does not happen automatically. Rather, it is by facilitating this support that a manager—who we call a "supportive supervisor"—enables the program to be a success.

The providers whom you will supervise are effectively on call 24 hours per day, 7 days a week – and at times, you may be, as well. In their correct focus on delivering health services properly, these front-line health workers often do not have time to worry about these other aspects of a health system which are critical to success.

The concept of supportive supervision is to place within the health system individuals whose purpose is to coordinate the aspects of the health system which support front- line health workers (such as CHWs) in service delivery. A supportive supervisor's job is to identify everything that his or her CHWs need to succeed—including continued training, supply of medicines, easy mobility, emotional support, regular and adequate pay, identification of mistakes, etc.—and ensure that these supports are in.

3. Administrative function

Supervision is a key part of management skills and in turn, also requires strong management skills. Being a good manager allows you to lead a team that can help many more people than you otherwise could by yourself. Managing a team of people involves helping them organize and prioritize their work, clearly communicating expectations, and making people feel valued – that is, making sure they know that their personal contribution makes a meaningful difference to the overall goals of the project.

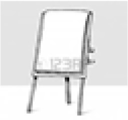

Activity two: Methods of supervision in community health services (40 minutes)

1. There are several methods of supervising the work done by CHWs; does anyone know what they are? Ask some members of the group to describe how they do their regular supervision. Ask what the last supervisory activity they did.

[1] Source Millennium Villages 2012.

2. Ask the other participants if they have different experiences of doing supervision. Was it one-on-one supervision or group supervision? If not, suggest #1 (one-one-one supervision), and then ask, can you think of any other types? Write down the methods of supervision on a flip chart.
    - Group supervision
        - By supervisor
        - By peers
    - One on one supervision
    - Home visit supervision
    - Spot checks

Ask the group: What do you think each of these methods of supervision mean? Do you do them? Can you explain what they mean? (If not, give explanations). Which sort of supervision do you do the most? Refer to the facilitators notes below to confirm the participants have identified each type e.g. Group supervision: do you do group supervision currently in your regular work? How do you do it? How often can you do it? Who comes? How is it organized? Give reflection on the techniques the respondents give.

Feedback information from the facilitator's notes below about the types of supervision where there are gaps. Spend the most time discussing group supervision and re-iterate to participants that this is the priority type of supervision for this training. During your discussion, write the following information on a flip chart:

Can be assessed in group supervision[2]

- Adverse events – emergency referrals, deaths
- Data on CHW inputs (coverage, visits, timing)
- Data on counselling health outcomes, tallying
- Current technical difficulties, trouble shooting, problem solving.
- Discussion of commonly identified barrier to health in communities.
- Soft skills within a group setting
- Observation of service delivery (simulations of practice)
- Health knowledge

Cannot be assessed in group supervision

Actual community practices of the CHW
Accuracy & reliability of data reported by the CHW
Solution: in this event a community health committee could track the progress of the community health service providers. The case evaluation assessment (see REACHOUT supervisor tools) can be simplified for conduct by a non-skilled literate community health committee member.

---

[2] Source World Vision 2013 Timed and Targeted Counselling

Facilitator's notes: Methods of monitoring community health services

Group supervision – is when multiple community health service providers gather to meet with the supervisor in either the health facility or a village. Meetings usually include regular supervisory activities (collecting data, discussing problems, and continuing education) in a group rather than individually. Ideally groups should contain a maximum of 15 people and meet monthly.

It is often the time when providers realize how they can help and support each other. Group supervision provides a rich forum for providers to share their unique experiences and challenges. Group supervision is beneficial to supervisees in that it provides a greater range of feedback, support, challenges and viewpoints. Supervisors should make arrangements to provide at least one monthly group supervisory meeting at the Health facility. Group supervision or debriefing is a mechanism for mitigating burnout among service providers in the helping and community work. Community health service providers face burnout as a result of interacting with clients in the course of their work. Support supervision or debriefing is recognized as a key approach for strengthening the quality of all aspects of community health service delivery, through working with community health service providers to identify areas of improvement and continuous professional development. Supportive supervision helps to maintain optimal provider performance and hence facilitating the delivery of high quality services.

The advantages of this method is that it takes a team approach which has been shown to be more effective for many contexts, it relieves the 'solitude of working' and helps community health service providers to feel like part of a bigger process and working towards common goals. The notion of 'peer pressure' is relevant too, as public presentation of data and progress amongst team members, can lead to public recognition of their efforts and is a disincentive for low performance. From the groups, typically more competent members will emerge, who can be strengthened to provide additional support to the community health service providers – become 'lead providers' over time, taking on roles such as data audit / support, trouble shooting, and conducting observational assessment or case evaluations in the community (after their skills are well developed) [3].

The peer-to-peer learning process which emerges during group approaches also has a huge value add to the project. Over time, community health service providers will become true experts in delivering services and many will encounter and overcome problems for themselves using local knowledge. This peer support may be a better source of solutions for overcoming cultural and behavioural barriers to health than that of the supervisor themselves.

Objectives of Group Supervision[4]:

---

[3] Source World Vision 2013 Timed and Targeted Counselling

[4] Source: Crigler et al 2013

Provides

1. a direct link between CHWs and the health system (protocols, guidelines, monitoring of quality)
2. supplies, drugs, and equipment
3. collection of information
4. group support for the CHWs

Key implementation considerations:

- Easy model to implement
- Supervisors need time to plan meetings, meet supervisees
- Proximity of communities.
- Method to support and measure success of individual CHWs.

Peer supervision – A group of providers who live close to each other and are at the same level (same job title) can meet together to share experiences or do problem solving activities and discussion. This is particularly important if the CHEW has more CHWs to supervise and is not able offer to offer group supervision to all of them at least once in a month.
Leaders for these groups need to be selected and mentored by the CHEW on how to do this and to feed back to the CHEW. If there are problems that cannot be solved these peer leaders feed back to their coordinator/supervisor. Peer supervision needs to be reported/minuted and submitted to the CHEW. If a CHEW has several peer CHWs he/ she can make plans to meet with them in a group supervision to not only discuss their work as CHWs but also their work as peer CHWs
Sometimes peer supervision can be negative and does not grow. If the groups are just complaining and not trying to find solutions then the CHEW needs to guide the peer CHWs on how to make it more supportive. The CHEW should also make a point of visiting the peer led groups at least times annually.

One-on-one supervision -usually is considered as traditional supervision - is when the supervisor and the supervisee to meet to discuss and provide support to make sure that the supervisee feels equipped and happy to fulfil their job effectively. The meeting needs to foster a culture of open discussion where supervisee has the time to discuss any concerns, worries and constraints within their role and the setting as a whole. The key elements of one to one supervision are coaching, training, personal development and the focus on their well-being. It should also be a two way process that enables both parties involved to developing a positive and mutually supportive discussion and on-going development plan. This strategy, however, is ideal in contexts with sufficient numbers of qualified and available supervisors

Home visit support supervision. Home visit support supervision focuses on issues such as **capacity building, staffing, logistics and supplies, data management, linking community health service providers and health facilities** etc. CHEWs should accompany or visit community health service providers in the field as they undertake their home visits to provide supervision on a monthly or quarterly basis. Supervision tools should be utilized to check and address all the service quality issues. Observed practice using a supervision checklist is a mechanism of providing instant feedback to community health service providers on a community health service session they had just conducted in a home or in the community. Observed practice is conducted by the CHEW

(supervisor), with the consent of the client/beneficiary. The supervisor, using a structured tool, is present during the session and gives feedback at the end of the session, based on what they had observed. Where capacity gaps are identified a number of corrective measures including immediate feedback, refresher trainings during group supervision or other capacity building opportunities including mentorship can be employed. Supervisors should ensure linkages between the community health service providers and the health facilities through navigating and aligning both clinic and community based priorities through regular interaction with both clinic and community-based staff.
Home visit supervision helps the supervisor to:

- Build a relationship with community health service providers
- Provide regular feedback and continuously help them improve their skills
- Collect data on how the CHW program is going
- Identify and troubleshoot logistical challenges
- Give the community health service providers an opportunity to alert their supervisor to problems with the program
- Motivate the community health service providers to do their job
- Provide moral support for the inevitable challenges of rural health care
- Reinforce the link between the clinic and the CHW and their community

What concerns may emerge under the administrative function for community health service providers?

Spot checks: This is usually a type of direct observation: (Look, listen and learn-3L's). It means systematically observing objects, events, relationships or people's behaviour, listening to what people talk about in an emotional way (excitement, anger, fear and concern), and learning and recording these observations in an organized manner. One does this by conducting transect walks. A checklist is necessary to ensure completeness of observation, based on the indicators that can be assessed through this method. The quality of observation can be improved by participating with the community in their activities. The supervisor walks from point A to B across the community often with the community health service provider. It is essential - in order to maintain trust - that community health service providers are informed that supervisors may occasionally perform spot checks.

Activity three: Group supervision practice session (40 minutes)

Start with an introduction to the group supervision exercise. Inform the group about the following issues:

- Every time you organize a group, you need to introduce the norms that you agreed in your first supervision group, remind the participants that the issues and situations you discuss are confidential, but what you learn is not confidential.
- Supportive, educative and administrative issues all come up in the group. As they come up you will address with them. A supervision group does not need a rigid structure, but is a structure that responds to the support needs of the supervisees and deals with problems as they come up, these will create opportunities to teach, train and role play. Sometimes issues will come up that you need to have training about at a later date.

- Although the group is a responsive environment, it is important to plan in advance if there are some important issues you need to feed back to the group. The first part of the group can be an unstructured session: Ask the CHWs for their experiences – how did you deal with a situation? How would others have done it? How can we improve on this?   If there are educative issues that come out then you have an opportunity to deal with these. This may take the majority of the time of the supervision.
- Make sure you leave adequate time after the supportive discussion session to cover any other issues on your agenda that need to be covered
- Remember, if there is a question you do not know the answer to, don't worry, you can say you will find out for the next session.

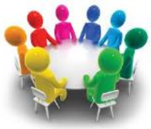

Activity three: Group activity – Supervision group role-play (40 minutes)

1. Ask the participants to get into pairs and take 10 minutes to plan a 30-minute supervision session, that will take them thirty minutes to role-play as supervision group. Allocate two of the groups to include an educative element for their supervision, two groups will focus on discussing group experiences of the last month, two groups will include an administrative element in their supervision.
2. Tell them you are not going to guide them how to plan it, but that they will learn from each other and there will be feedback after each mock supervision group. In future they will usually lead the supervision group alone, but for this training they will do it in pairs.
3. After 10 minutes planning the group back together, ask for volunteers for the first pair to supervise the group. Ask all the other participants to role play as CHW supervisees. Arrange the whole group in a circle.
4. Have the volunteers practice while completing the group supervision tool
5. The supervisors lead the group for 30 minutes asking the CHWs members to participate fully.
6. At the end of the group ask each of the two supervisors to give feedback:
    a. What is your general feeling about the experience of leading the group generally
    b. What did you find difficult?
    c. How did you find the group supervision tool?
7. Ask the participants to give feedback:
    a. What did the supervisors do well?
    b. What did not go so well?
8. Facilitator gives feedback – start with positive feedback
9. Ask the next group to do their supervision group, repeat process of 2 to 6 for all pairs of supervisors). After half of the groups have performed give some more detailed feedback (see notes below), then continue with the rest of the groups.

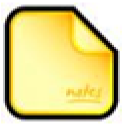

Facilitator's notes

Examples of good practice: (it is up to the judgement of the supervisor how to run the group in a way that suits their supervisees, the examples below are for the supervisor to consider integrating into their session.

Introductions:

1. Consider starting by thanking the participants for coming, for their hard work, and for arriving on time. Remind them that everyone has a voice and that the best way to help each other is by sharing experiences. This is an opportunity to talk with each other and learn from each other and for us to listen and find ways to support you. Tell them what time the
2. Re-state the group norms (agreed in previous meetings), for example:
    a. Be punctual
    b. Non-judgemental environment
    c. Respect each other
    d. No right or wrong answers
    e. Work together as a team in which everyone agrees that they will contribute.
    f. Confidentiality – the issues we share today are confidential, and what we learn is not
3. Agenda. Consider telling the group the running order of the meeting. It may not always be realistic to follow the order of the agenda closely, but ensure that all the key activities are undertaken and pay close attention to timing of the activities. An example agenda may be:
    a. Discuss matters arising from the last meeting and action points achieved
    b. Sharing experiences and problem solving
    c. Learning session / refresher training
    d. Data feedback and reporting on activities for the month
    e. Feedback from issues arising at district level and updates from coordinators

Mutual support and sharing experiences: This session may make up the majority of the meeting and needs to have clear action points arising from it.

1. Sometimes it works well to ask everyone to share a positive experience in their work since the last meeting. Congratulate them where appropriate and give insight to how this is important if appropriate. Supervisors can give a brief feedback and summing up on the good work. Consider asking them if there is anyone who wants to thank their peers for any support they have received from them, or if they have been shown any appreciation from the community.
2. Problem solving. Give all participants some time to present challenges they have had since the last meeting. When the first person presents a challenge, be sympathetic thank them all for sharing and ask if other people have had similar problems. Supervisor should take clear notes (including action points). Try to think about educative supervision elements that can be incorporated into the discussion on challenges. Start discussing how the problem can be solved, and inviting other participants to suggest solutions too. If participants are reluctant to start contributing ask some questions like, "does anyone have anything to share?" "has anyone experienced a community member who has refused to accept your services (e.g. immunization or facility delivery)?", "has anyone experienced any challenges with referrals to the health facility?"
3. Ensure that each participant has contributed, ask those that have kept quiet to share their

experiences, you can explain it is for the benefit for everyone that we learn from each other.

4. For each problem give a clear answer of how the solution may be found, and record action points, with a timeline if possible.

Communication:

1. First impressions are important, try to build trust from the start. Make it clear that you are there to listen and support
2. Names: Try to know the names of all participants and address them by their name so they know they are recognized.
3. A supervisor can expect to hear criticism of him/her or the system. Try not to be too defensive or to pass the blame, but try to find ways to find realistic solutions, or that you will consult elsewhere to bring a solution next time. It is difficult for people (especially volunteers) to stay motivated if realistic solutions are not sought.
4. Encourage and praise participants when they demonstrate good practice.
5. Participants may interrupt with an issue that you don't want to deal with right away. Do not block their suggestion too aggressively. Thank them for their contribution and say you would like to return to it in a while – the participant is welcome to remind you about it later if you like.
6. Thanks: Remember to thank participants for their contribution.
7. I and We. Even if you are supervising the group alone it is a good idea to use the term 'we' instead of 'I'. As a supervisor you do not stand alone, there are other supervisors, coordinators, and health system members which you are an integral part of.
8. Remind participants they also have a responsibility to try to encourage and motivate each other.
9. Try not to complain, the supervisor may feel overworked, but try not to use other commitments as an excuse for not performing as the participants expect.
10. Be aware of contradicting yourself or promising things that are not realistic, consider your responses and try to stay composed even in the face of challenges and criticism.
11. Sometimes there is conflict in the group, encourage the participants that we will try to come to a consensus.
12. Take issues seriously, do not appear too relaxed.
13. Try to avoid an accusative tone of voice if people are making mistakes as this will make participants defensive.
14. Dress appropriately: don't look too formal like a manager, but look approachable. Remember the SONER body-language advice.

Report: Take notes of proceedings in the Group Supervision tool which should also have action plans for activities to be followed up.

At the end of the day review the EASY/DIFFICULT skills which are displayed on the wall. Discuss how many of the 'difficult' ones can now be moved to the 'easy' side.

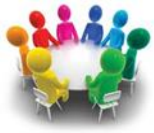

Activity four: Conducting One-on-One Supervision (20 minutes)

- 4 CHEWs to each conduct one-on-one supervision on a fellow CHEW with the class as audience (20 minutes each, practice with the one-on-one tool)
- The 4 CHEWS, their participants and class share with the class their thoughts on their experience and use of tool.

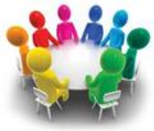

Activity five: Conducting home visit supervision (20 minutes)

- Facilitator to divide the participants into groups of 4
- Provide the home visit supervision tool to each group and ask the groups to present back what they think of the components of the tool e.g. relevance, what needs to be added
- Ask each group to present their responses to the larger team and have a discussion with the group

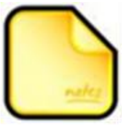

Facilitator's notes

Conducting a Supervisory Home Visit:

- Formally schedule a visit with a specific date and time
- Ensure you are introduced to the household while you are present, and the purpose of your visit with the CHW
- Allow the CHW to carry out the visit as if you were not present
- Identify whether the CHW is conducting all of his/her tasks, according to the stated responsibilities
- Assess how the CHW is conducting his/her tasks – quality of service, etc.
- Thank the family for their time at the close of the visit
- Provide feedback to the CHW in a quiet, private place, including both positive and critical feedback for improvement
- Identify with the CHW key areas for improvement that will be reassessed in the following observational visit
- Assess the CHW's data forms for completeness and accuracy (based on what activities were observed in the visit)
- Ask the CHW if there were any outstanding issues that s/he would need support on during household visits
- Schedule a time and date for the next observational visit

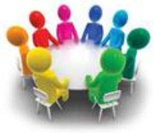

Activity six: Conducting supervisory spot checks (20 minutes)

- Facilitator to divide the participants into groups of 4
- Provide the spot checks tool to each group and ask the groups to present back what they think of the components of the tool e.g. relevance, what needs to be added
- Ask each group to present their responses to the larger team and have a discussion with the group

Module 4: Community engagement

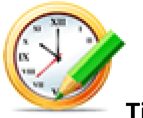

Time: 2hrs

By the end of this module, the participants will be able to:

- Define community engagement
- Understand the process of community engagement
- Describe community dialogue and the steps for community dialogue

Session 4.1: What is Community Engagement

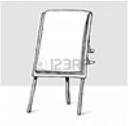

Activity one: Brainstorming the term community engagement (10 minutes)

- Facilitator to brainstorm the definition of community engagement
- Write the responses on the flip chart and have a discussion with the group
- Fill in the gaps using the notes below.

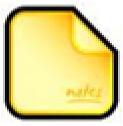

Facilitator's notes

Community engagement' is a planned process with the specific purpose of working with identified groups of people, whether they are connected by geographic location, special interest, or affiliation or identify to address issues affecting their well-being. The linking of the term 'community' to 'engagement' serves to broaden the scope, shifting the focus from the individual to the collective, with the associated implications for inclusiveness to ensure consideration is made of the diversity that exists within any community. Community engagement can take many forms and covers a broad range of activities.

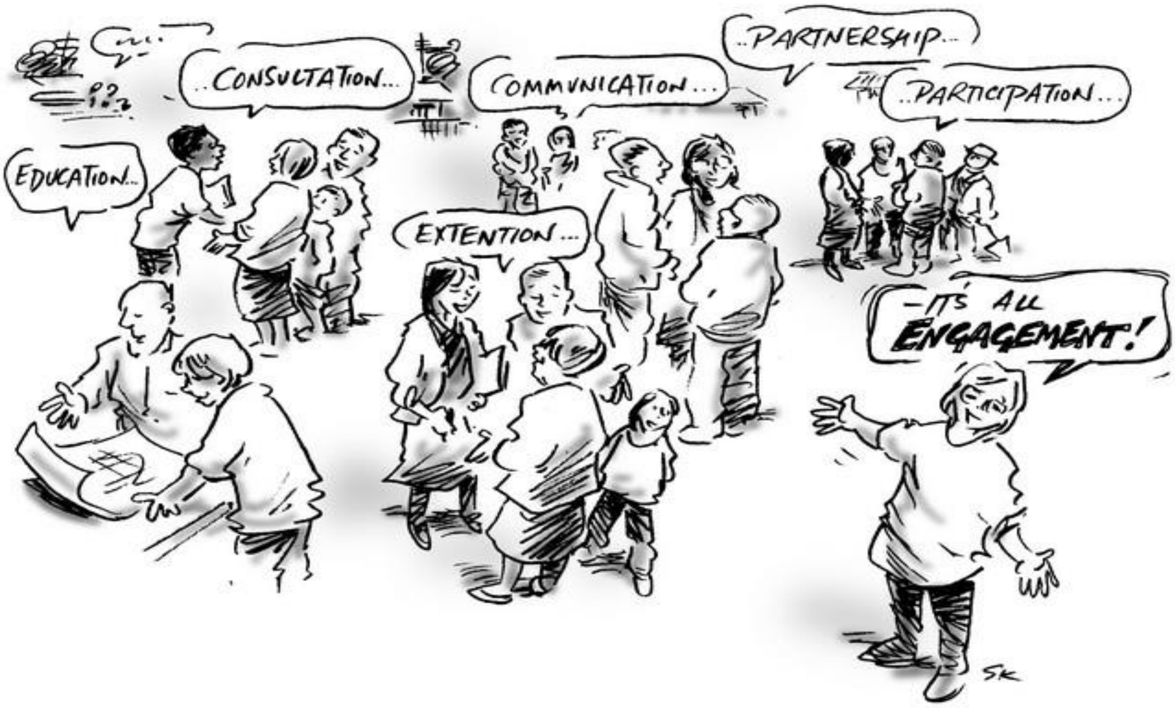

Community engagement works best where it is a process that enables relationships and trust to build and strengthen over time. Engagement events should be planned and designed with this in mind. Community groups may want to participate at a range of levels:

- Providing advice at the planning and designing level
- Undertaking some aspects of the engagement
- Delivering projects to meet some of the outcomes

Session 4.2: Role of Community Engagement in Supervision of CHWs

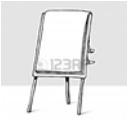

Activity one: Importance of community engagement for supervisors (10 minutes)

- Facilitator to brainstorm importance of community engagement for CHEWs in their work
- Write the responses on the flip chart and have a discussion with the group
- Fill in the gaps using the notes below.

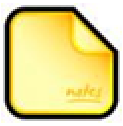

Facilitator's notes

CHEWs have an important role of ensuring that CHWs deliver quality services to the community they serve. Health services assessed to be of high quality according to the provider-defined criteria is far from being ideal if the client is dissatisfied with it. There is evidence that involving the community in health solutions can help improve the quality of

health care and even patient outcomes. The community members need to be involved in defining what quality service is and there is also need for feedback from the community about the kind of services they receive or would want to receive. This can help CHEWs in planning and making decisions.

Community engagement is therefore important and can assist the CHEW in the three functions of supervision as follows:

1. Educative (formative)— identify capacity building areas which need reinforcing from feedback given by community members
2. Supportive (restorative)— make the community members portray acceptance and appreciation towards CHW work
3. Administrative (normative)— find out whether CHWs are visiting their designated households as required

Community engagement is important for CHEWs in the following ways:

- Community can be informed of policy directions of the government e.g. informing them of what Community Health Strategy entails.
- Community can be consulted as part of a process to develop government policy, or build community awareness and understanding towards the same e.g. making them understand and support the roles of both the CHEWs and CHWs.
- Community can be involved in a range of mechanisms to ensure that their issues and concerns are understood and addressed by CHEWs e.g. by getting feedback from them on work done by CHWs
- Through engagement the community will be considered in the decision-making process e.g. in dialogue days, and this will go a long way in encouraging their support towards initiatives such as action days
- Collaborations can be developed with the community by developing partnerships to formulate options and provide recommendations to problems that affect them e.g. coming up with recommendations during dialogue days
- Community can be empowered to implement and manage change e.g. through involving them in action days and involving them in advocacy.

Session 4.3: How CHEWs engage Communities in Community Health Services

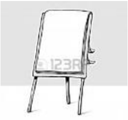

Activity one: Brainstorming Community Health Committees (CHCs) roles (20 minutes)

- Give each participant a list of the roles of CHC members.
- Divide the group into pairs.
- Ask the groups to consider each CHC role and discuss the following questions: Are they

important? Are they useful? Have they been regularly taken up by the CHC members in their areas - Ask them to come up with example situations that illustrates the point.

- Ask the members to share their discussion with the larger team (try to keep these presentations as brief as possible)

Facilitator should review the results:
Do the participants feel the same way about the roles of CHCs? Are there roles that have been agreeably neglected by CHCs and why? Spend some time discussing with the participants about ways that they feel CHCs can be supported in their roles and by who

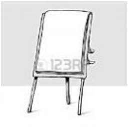

Activity two: Community Health Committees (CHCs) vs. CHEWs roles (20 minutes)

- Hand the list of CHEWs' roles to the participants
- Ask the participants to go back to their groups of pairs and refer to the list of their roles and that of the CHEWs
- Ask each group to discuss the following questions: Which of their roles and that of the CHEWs complement each other? Which roles do they feel overlap.
- Ask the members to share their discussion with the larger team (try to keep these presentations as brief as possible

Facilitator should review the results:
Do the participants feel the same way about their analysis of their roles and that of the CHCs? Which complementary roles of the CHEWs and CHC members have been less or more challenging to perform together? Are there overlapping roles and how do the CHEWs cope with these? Spend some time discussing with the CHEWs about ways that they feel they can work with the CHCs better.

Session 4.4: Community Dialogue Days

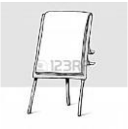

Activity One: Role play and Sharing Experiences on Dowry Negotiation (20 minutes)

1. Ask the participants to volunteer and show case how dowry discussions are done in their community. Get two families, one for the groom and the other for the bride including aunties and uncles from the plenary, let them hold dowry negotiations for 10 minutes. Insist they do it as per their culture
    - Ask the participants whether they saw any dialogue.
    - Ask the participants to share their experiences on Dowry Negotiation, using the following questions:

- How many people were involved in dowry negotiation?
- Was there a common understanding among people involved?
- What action did the dowry negotiation lead to in the end?

2. Ask the participants the characteristics of a dialogue. Summarize with the key points below.

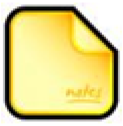

Facilitator's notes

Key Points on Community Dialogue

Community Dialogue is:

A mutual continuous exchange of views, ideas and opinions about an issue or a concern

"Dialogue is one of the most important strategies in the Community Strategy to make people's behaviour and community change. Organizing and facilitating Community Dialogue is done by the CHC, while the mobilization is done by the CHWs.

Characteristics of Community Dialogue

1. It involves interactive communication between two or more parties, aimed at reaching a common understanding on issues for the purpose of taking action

2. Dialogue meetings are held quarterly (4times in a year) and members who participate include CHWs, CHEWs, CHCs, sub county Health Management teams, partners and members of the public

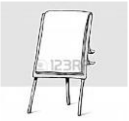

Activity two: Conducting Community Dialogue Days in Community Health Services (20 minutes)

- Ask two participants to each volunteer and show case how they carry out community dialogue days in their community. Get two volunteers at each instance with the rest of the participants role playing as attendees.
- Ask the participants whether they saw any dialogue at the end of each role play.
- Ask the participants to share their experiences with Community Dialogue Days using the following questions:
    - How many people are involved and what criterion is used to invite them?
    - Who mobilizes people for attendance?
    - What preparations are made before dialogue days?

- What informs the agenda of the dialogue day?
- How are solutions/ recommendations arrived at?
- How are action points dealt with?

- Summarize with the key points below.

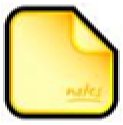
Facilitator's notes

Key Points on How Community Dialogue can help

- Seek to satisfy everyone's needs
- Win-win solutions
- Find other's strengths
- Look upon others as a friend
- Open up the communication
- Ask questions and show that you want to learn
- Create energy by listening actively, asking, inspiring in a positive way, and involving
- Seek more solutions

Steps in carrying out community dialogue days

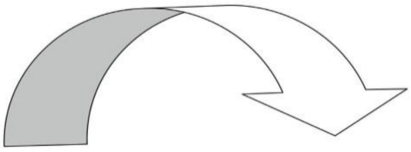

1. **Setting the stage**: Making an entrance into the community leaderships and community structures. (CHC, HFMC, Prov. admin)
2. **Organized groups identification and mapping**: Knowing which organized groups exist in the community, where they are and what they do.
3. **Making visible the unexpressed needs**: Helping the communities to identify the most important needs and how to address them.
4. **Making organized groups action plans**: Based on the needs and the current status, the community will make plans on how to achieve what they want to be in the future.
5. **Ensuring sustained dialogue and results for development**: Linking communities and services, supporting the organized groups to carry on by themselves

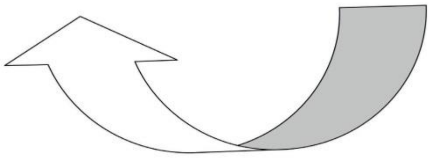

Activity three: Facilitating Community Dialogue (10 minutes)

Mini-lecture

The LePSA method of adult learning emphasizes participatory methods of learning, in recognition of

the fact that adults have their peculiar characteristics and expectations. It is one of the best methods of helping communities to learn from each other. It is also referred to as a problem solving process.

LePSA stands for:
Le- Learner centered
P- Problem posing
S - Self-discovery
Action oriented
It involves the use of a starter, followed by "SHOWeD" questions.

What to consider when preparing a starter:-

- Deal with the theme on which the community has strong feelings
- Show familiar scenes in everyday life
- It should stimulate interest and move emotions
- Deal with one theme
- It should be simple, clear, and visible
- Avoid distracting details
- A code should raise questions but not provide solutions
- It should portray one problem.

The “SHOWeD” questions are:-

- What did you SEE or hear? (describe the situation depicted by the starter)
- What was HAPPENING? (Interpret the situation and identify the problem)
- Does it happen in OUR community/experience? (relevance)
- WHY does it happen? (identify and analyse the root cause)
- What similar EXAMPLES can be given in our area? (Extent! Self-discovery)
- What can we DO about the situation? (solutions, resources, action plan)

The SHOWeD questions’ are used to guide discussions on a particular problem for the purposes of encouraging the problem solving process. The participants then prioritize and appraise the doable action

Session 4.5: Community Dialogue Can Translate the Problem to Become a Community Concern

Activity four: Brainstorming on initiating dialogue sessions through utilisation of the community chalk board (10 minutes)

- Ask the participants “How can the chalk board generate issues for dialogue in the community?” Allow response from the plenary and discuss for 5 minutes.

Facilitator’s notes

The trend of the indicators on the chalk board can guide the dialogue by the community members asking themselves why the numbers are increasing or decreasing based on their community experiences

Linking community dialogue to action day

Like dowry negotiations lead to a wedding day, so does a dialogue day lead to an **Action**.

The community should make an action plan during the dialogue day, and the action plan is implemented on the action day.

This means action days are held as per the action plans. The frequencies of the action days will be determined by the priority needs of the community

Module 5: Monitoring and Evaluation in Supervision

Time: 1 hr

By the end of this module, the participants will be able to:

1. Describe the supervision data management process
2. Demonstrate ability to effectively manage community health services data
3. Demonstrate ability to implement activities in each of the supervision components
4. Describe different methods of monitoring CHWs work
5. Demonstrate ability to utilize supervision monitoring tools

Activity one: CHEW's role in data review (15 minutes)

- Facilitator to brainstorm with the group the supervisor's role in data collection
- Write the responses on a flipchart
- Fill in gaps and add using the information below.
  - If on a paper based system: Collecting his/her community health service providers' paper forms weekly and regularly submitting to the office for data entry.
  - If on a paper based system: Collecting CHW reports from the office to distribute and review with community health service providers weekly.

Facilitator's notes

- The supervisor should be able to develop a schedule to discuss priority topics such as emerging diseases, upcoming health events, and maintenance of continuum of care, and deliver refresher training on these according to local guidance, in group supervision sessions.
- The supervisor should be able to identify with health facility in-charges and community health service providers the best way of having community health service providers support the health facility and vice versa.
- The supervisor should be able to ensure that community health service providers are kept up-to-date on new health facility activities, promotion as well as scheduled health days and fairs that they are required to assist in.
- Supervisors will be able to follow up on community health service provider data collection, ensuring regular, complete collection at each household visit to ensure data is being collected regularly and at each household visit.
- If the community-based data system uses a paper-based platform, the supervisors should be able to regularly follow up on the compilation of reports by CHWs on a monthly basis for

submission to the office for data entry

Data Analysis

Data analysis is a process of gathering, modelling, and transforming data with the goal of identifying performance gaps, highlighting useful information, suggesting conclusions, and supporting decision making. Through data collection, variances can be detected thus a reliable way of monitoring quality. A plus for data analysis include the fact that it is accurate, data can be analysed using different indicators like by age, sex, uptake, resources, type of illness etc. At the same time, identifying problems is easy. Setback for data analysis is that it is time consuming and requires a person with data analysis skills.

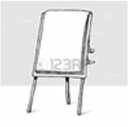

Activity two: Data Analysis (45 minutes)

1. Give each participant a filled 514 log book and a blank 515 summary tool
2. Ask the participants to fill in the information from the 514 log book to the 515 summary tool
3. Check if the participants are filling in correctly and if they are able to identify any gaps from the data
4. Ask participants to share how they utilise the data; (what do they understand from the data and who do they report to. Do they ever make any changes based on the data?)
5. Ask how they address the gaps; do they give feedback to the community health service providers? The CHEWs supervisors? When?
6. What is the information flow? Who do they give the 515 summary tools? Who fills in the chalkboard (516)?

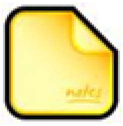

Facilitator's notes

Supervisors need to be able to identify any gaps emerging from the data collected by the community health service providers, address the gaps for example by working through the gaps with the community health service providers or giving feedback. The supervisors need to be know whom they report to and how the information they get is used to address any emerging gaps within the community and ensure that results are observed.

In many systems, data collection sheets are the only form a supervisor is required to submit following supervision, i.e. there is a string emphasis on data collection, however, once it is collected it does not get used appropriately. Mostly, it is a one-way data flow and the community health service provider may experience little benefit from the data collected.

Supervisors should know what threshold levels for performance indicators are considered good, needing improvement, or poor and able to assess and share the feedback in order to identify the weaknesses and develop an action plan for the community health service provider to improve in that area.

Supervisors need to promote quality of the community health service provider's work over the quantity e.g. looking at how many people in the houses took up the services, what were the outcomes of the treatment etc. rather than how many houses were covered or number of treatments given. Supervisors needed to support the community health service providers by looking at issues that make them work smarter and with greater effect than issues that make them work harder.

Module 6: Ethical considerations in supervision

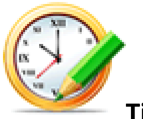

Time: 1 hr

By the end of this module, the participants will be able to:

1. Define the code of ethics in supervision
2. Demonstrate capacity to support supervisees in resolving ethical issues

Session 6.1: Code of Ethics in Supervision

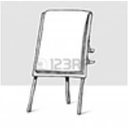

Activity one: Exercise (1 hour)

- Participants to share ethical dilemmas and how they resolved them.
- Facilitator to engage the group in discussions and offering feedback

Activity two: Scenarios

- Facilitator to divide the group into 4-5 participants
- Each group discusses ethical scenarios and reports in the larger group.
- Facilitator lays emphasis on how to resolve ethical dilemmas
- Facilitator then introduces the code of ethics as guided by the notes below

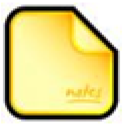

Facilitator's notes

<u>Code of ethics</u>

Is a set of statements about appropriate and expected behaviour of members of a professional group and, as such, reflects its values. The purpose of code of ethics is to establish and maintain standards for supervisors in their supervision work with the community health workers, hereinafter referred to as supervisees, and to inform and protect supervisees seeking supervision.

Confidentiality

The content of supervision is maybe highly confidential. Supervisors must clarify their limits of confidentiality

Safety

All reasonable steps must be taken to ensure the safety of supervisors and the community health service providers during their work together.

Effectiveness

All reasonable steps must be taken by supervisors to encourage optimum levels of practice by trainee supervisors.

Contracts

The terms and conditions on which supervision is offered must be made clear to work within the limits of that competence. This includes having their own supervision work supervised.

Competence

Supervisors must take all reasonable steps to monitor and develop their own competence and to work within the limits of that competence. This includes having their own supervision work supervised.

<u>Code of practice</u>

This code of practice is intended to give more specific information and guidance regarding the implementation of the principles embodied in the code of ethics.

Issues of Responsibility

The primary purpose of supervision is to ensure that the supervisor is addressing the needs of the CHW:

Supervisors are responsible for helping Supervisees reflect critically upon that work.

Supervisors and Supervisees are both responsible for setting and maintaining clear boundaries between working relationships and friendships or other relationships.

Supervisors must recognise the value and dignity of Supervisees and Clients as people, irrespective of origin, status, sex, sexual orientation, age or belief.

Supervisors should not exploit Supervisees financially, sexually, emotionally or in any other way.

Supervisors are responsible for establishing clear working agreements, which indicate the responsibility of supervisees for their own continued learning and self-monitoring.

Both are responsible for regularly reviewing the effectiveness of the Supervision arrangement, and changing it when appropriate.

Issues of Competence

Supervisors should continually seek ways of increasing their own professional development, including, wherever possible, specific training in the development of supervision skills.

Supervisors are expected to make arrangements for their own consultancy and support to help them monitor and evaluate their supervision. This includes having supervision of their supervision work.

Supervisors have a responsibility to monitor and maintain their own effectiveness. There may be a need to seek help and/or withdraw from the practice of Supervision, whether temporary or permanently.

References

Crigler, L., Gergen, J.& Perry, H. (2013). Supervision of community health workers.

International HIV/ AIDS Alliance. (2002). Advocacy in Action: A Toolkit to Support NGOs and CBOs Responding to HIV/ AIDS in http://www.iasociety.org/Web/WebContent/File/Alliance%20-%20Advocacy%20in%20Action.pdf

LVCT Training Institute. Training manual for training support supervision in HIV testing and counselling

Liverpool VCT, Care and Treatment and Regional AIDS Training Network. (2012). Quality Management Training for HIV Testing and Counselling Services

Millenium Villages (2012). Milleniuum villages project: CHW supervision trainer's manual.

World Vision. Timed and targeted counselling for health and nutrition.
