# Supplementary material for: Strengthening referral systems in community health programs: a qualitative study in two rural districts of Maputo Province, Mozambique
Source: BMC Health Serv Res. 2019 Apr 29;19:263. doi: 10.1186/s12913-019-4076-3 (PMC6489304; doi:10.1186/s12913-019-4076-3)
Supplement: Supplementary file 2 — Referral In-depth Interview Topic Guide 2. (DOCX 15 kb) [file 12913_2019_4076_MOESM2_ESM.docx]

**Referral In-depth Interview Topic Guide**

1. **Reason for referral**
2. Can you tell me what happened when you last saw the CTC provider?
3. Why were you referred?

*Probe: what for (as per context)*

1. Where were you referred to? (name/location of facility)
2. **Decision**
3. Did you go to the health facility you were referred to?
4. What reasons made you decide to go or not go to the facility after the referral?
5. **Probing reasons for referral decision**
6. Do you feel that you have the knowledge, money and skills to attend the health facility after the CTC provider refers you to go there? *Probe why?*
7. What are the advantages of going to the health facility when the CTC provider refers you to go there? *Probe why?*
8. What are the disadvantages of going to the health facility when the CTC provider refers you to go there? *Probe why?*
9. Do most of the people you know approve of you going to the health facility when the CTC provider refers you to go there?

*Probe: Who are the people that approve of you going to the health facility when the CTC provider refers you to go there?*

*Probe: Who are the people that disapprove of you going to the health facility when the CTC provider refers you to go there?*

1. What makes it easy for you to go to the health facility when the CTC provider refers you to go there?
2. What makes it difficult for you to go to the health facility when the CTC provider refers you to go there?
3. How likely is it that the nurse/ health worker at the health facility would be able to handle a problem after you have been referred? *Probe why?*
4. How difficult is it for you to access the nurse/ health worker at the health facility after you have been referred? *Probe why?*
5. How likely is it that you will have problems if you do not go to the health facility after having been referred? *Probe why?*
6. What shapes decisions about going to the health facility after you have been referred?

*Probe on: Cultural beliefs, religious beliefs, social issues*

1. **For patients who did not go to the health facility after having been referred**
2. Did you seek help or services elsewhere?

*Probe: into other informal providers depending on contexts, other private or public facilities*

1. **For all patients**
2. Thoughts on future decisions
3. Based on this experience of the referral process (not just the experience received at the referral site), would you choose to do the same thing if you were referred again by the CTC provider? *Probe: Why/why not?*

**Referral Focus Group Discussion – community level, Topic Guide**

1. **Problem description**
2. What are the main challenges getting health care in your community?
3. What are the main challenges with health services provided by CTC providers in your community?
4. Where is your first choice to go for health care? Why?
5. Are there places you won’t go to for health care? Why?
6. **Coordination and referral**

In what circumstances do CTC providers make referrals?

*Probe Has this changed? How? What do you think about the changes?*

How do people feel if the provider makes a referral to a health facility?

*Probe How is the quality of referral (adapt) services that are available in this health facility? How likely is it that the nurse/ health worker at the health facility would be able to handle a problem after you have been referred? Why?*

How could referral and coordination of health services be improved?

Where or from whom do you receive information about health services?

*Probe Has this changed? How? What do you think about the changes?*

What health information or education messages or activities are you aware of in the last year? What do you think about them? How is the quality of the information you received?

*Probe Has this changed? How? What do you think about the changes?*

Whose advice do you trust or take when making decisions about seeking health care? Has this changed?

There is something related to a referral system that we didn't have the opportunity to discuss and that you would like to share with us?
